# A dynamic, modifiable model for estimating cost‐effectiveness of smoking cessation interventions in pregnancy: application to an RCT of self‐help delivered by text message

**DOI:** 10.1111/add.14476

**Published:** 2018-12-05

**Authors:** Matthew Jones, Murray Smith, Sarah Lewis, Steve Parrott, Tim Coleman

**Affiliations:** ^1^ Division of Primary Care University of Nottingham Nottingham UK; ^2^ Community and Health Research Unit University of Lincoln Lincolnshire UK; ^3^ Division of Epidemiology and Public Health University of Nottingham Nottingham UK; ^4^ Department of Health Sciences University of York York UK

**Keywords:** Cost‐effective, economic evaluation, pregnancy, smoking, smoking cessation, tobacco

## Abstract

**Background and Aims:**

Previous evaluations of smoking cessation interventions in pregnancy have several limitations. Our solution to these limitations is the Economics of Smoking in Pregnancy (ESIP) model, which estimates the life‐time cost‐effectiveness of smoking cessation interventions in pregnancy from a National Health Service (NHS) and personal social services perspective. We aim to (1) describe how ESIP has been constructed and (2) illustrate its use with trial data.

**Methods:**

ESIP links mothers’ and offspring pregnancy outcomes to estimate the burdens of smoking‐related disease they experience with different rates of smoking in pregnancy, both in pregnancy and throughout their life‐times. Smoking rates are inputted by model users. ESIP then estimates the costs of treating disease burdens and also mothers’ and offspring life‐years and quality‐adjusted life years (QALYs). By comparing costs incurred and healthy life following different smoking rates, ESIP estimates incremental cost‐effectiveness and benefit–cost ratios for mothers or offspring or both combined. We illustrate ESIP use using data from a pragmatic randomized controlled trial that tested a smoking cessation intervention in pregnancy.

**Results:**

Throughout women's and offspring life‐times, the intervention proved cheaper than usual care, having a negative incremental cost of £38.37 (interquartile range = £21.46–56.96) and it improved health, demonstrating a 0.04 increase in incremental QALYs for mothers and offspring, implying that it is ‘dominant’ over usual care. Benefit–cost ratios suggested that every £1 spent would generate a median of £14 (interquartile range = £8–20) in health‐care savings.

**Conclusions:**

Economics of Smoking in Pregnancy is the first economic model to link mothers’ and infants’ costs and benefits while reporting cost‐effectiveness in readily‐comparable units. Using ESIP with data from a trial which reported only short‐term economic analysis showed that the intervention was very likely to be cost‐effective in the longer term and to generate health‐care savings.

## Introduction

Tobacco smoking during pregnancy remains a major global public health concern estimated to cost £23.5 million annually in the United Kingdom 1 and US$110 million in the United States; 2 the prevalence varies from 39% in Spain 3 to 23% in Canada 4 and 12–14% in the United Kingdom, United States, Australia and Germany 5, 6, 7, 8. Many mothers expose themselves and their offspring to both pregnancy‐related and long‐term risks from smoking 9, 10, 11, 12.

Economic evaluation is important for demonstrating the value for money afforded by programmes competing for scarce health‐care resources. Previous evaluations of smoking cessation interventions for pregnant women have been inconsistent, making comparison of findings difficult 13. For example, models have: treated maternal and infant health outcomes as mutually exclusive 14; provided outputs in ‘cost‐per‐quitter’ units which cannot be compared with other economic measures 15; not adequately justified the inclusion of morbidities 16; or have provided only limited allowance for uncertainty 17.

We describe the Economics of Smoking in Pregnancy (ESIP) model which we designed to address these limitations, by estimating the future health gains and treatment costs associated with both the mother and her infant up to the age of 100 years, using a UK National Health Service (NHS) and Personal Social Services (PSS) perspective 18. We also demonstrate how ESIP estimates the cost‐effectiveness of a within‐pregnancy cessation intervention by using the data from a recently published trial.

## Description of ESIP model

### Overview

The cost‐effectiveness of a smoking cessation intervention can be expressed as the ratio of the increased ‘per‐person’ costs of providing that intervention to the ‘per‐person’ health benefits that the intervention causes. In the short term, costs mainly comprise paying for intervention delivery; however, if an intervention promotes cessation and smoking‐related diseases occur less frequently, then longer‐term costs for treating these reduce. For women and infants and for different smoking rates in pregnancy, ESIP estimates the burden of smoking‐related disease in pregnancy and during their life‐times and calculates the health service costs incurred treating this. ESIP also estimates the potential life years that women and infants can expect before adjusting these into standard economic terminology, quality‐adjusted life years (QALYs). By comparing costs incurred due to different smoking rates, ESIP estimates how much an intervention costs or saves; similarly, by comparing healthy life which accrues following different smoking rates in pregnancy, ESIP estimates whether a cessation intervention provides health benefit; finally, these cost and benefit estimates are combined to generate cost‐effectiveness measures.

### ESIP components

**Figure 1:**
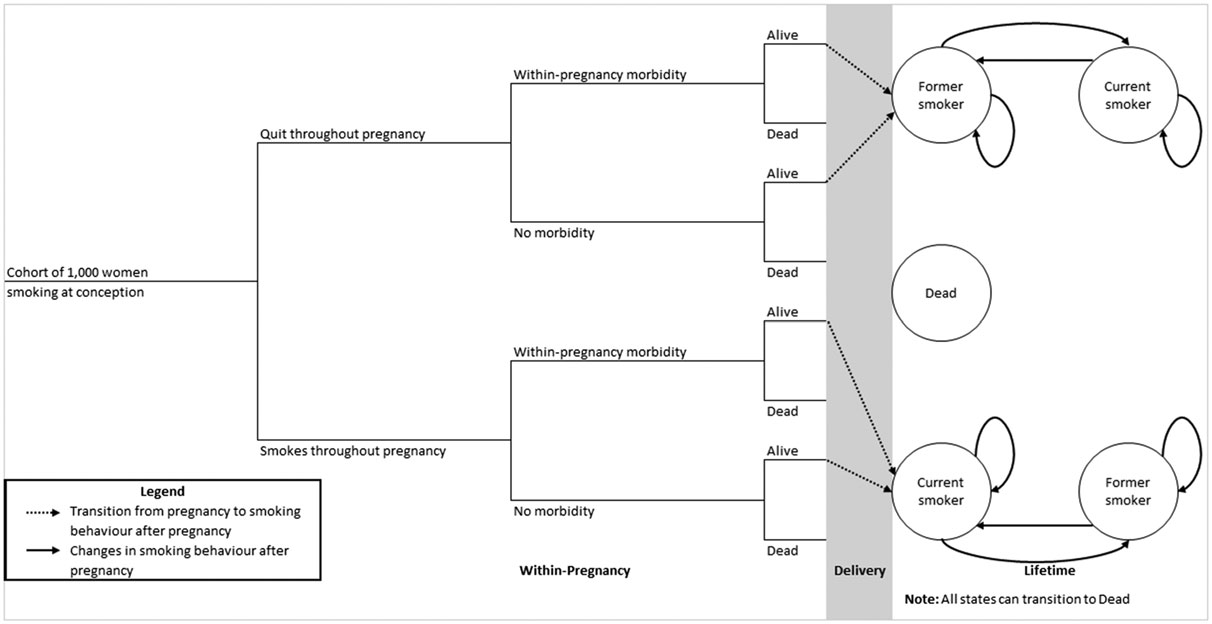
Simplified maternal model: women progress through ‘within‐pregnancy’ decision tree determining their ‘states’ on entry to life‐time Markov component

Figure 1 provides a simplified maternal model structure (full detail in Supporting information, Appendix S1). A hypothetical cohort of 1000 singleton‐pregnancy women who smoke enter a decision tree (left‐hand side), which estimates smoking‐related morbidity in pregnancy; the first branch of this tree is where smoking rates in pregnancy are entered into the model, affecting all model calculations. The tree determines women's smoking behaviour at childbirth, whether or not they survive pregnancy and whether live births occur. Next, surviving women enter a ‘life‐time’ Markov chain model component (right‐hand side) that predicts changes in their life‐time smoking behaviour and, dependent on this, determines their life‐time burden of smoking‐related morbidity and mortality. We defined ‘life‐time’ as modelling women's and infants’ outcomes until 100 years old or death.

**Figure 2:**
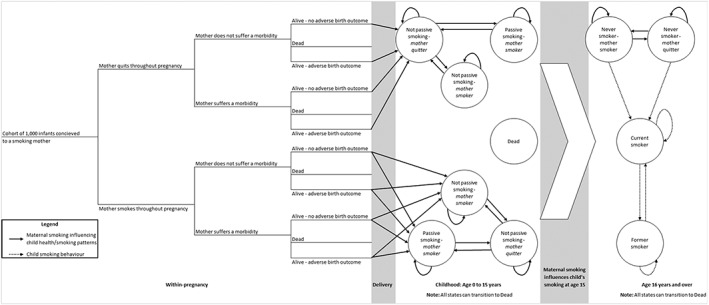
Simplified fetal and infant model: offspring progress through ‘within‐pregnancy’ decision tree determining ‘states’ for entry into Markov childhood and life‐time components

Figure 2 shows a simplified fetal model structure (Supporting information, Appendix S1). Fetuses conceived by virtual cohort women enter a decision tree (left‐hand side) which determines how many survive to birth and, of these, what proportion have low birth weights. Key parameters in the fetal decision tree (e.g. proportion of stillbirths) are generated in the maternal one; hence, both are linked and fetal pregnancy and birth outcomes are dependent on smoking rates entered into the maternal tree. Between birth and 15 years infants enter a ‘childhood’ Markov chain component (middle section) which estimates their burden of asthma, factoring in the impact of second‐hand exposure to maternal smoking, with smoking rates used, coming from the maternal life‐time model. However, not all women who smoke expose their child to their smoking, and this is allowed for (see Table S1 in Supporting information, Appendix S2). However, it is assumed that once a mother exposes a child to passive smoking, this behaviour would not change. This component also estimates children's rates of smoking uptake incorporating an allowance for the influence of maternal smoking 19. Finally, at 16 years children enter an ‘adulthood’ Markov chain component (right‐hand side) which estimates their life‐time burden of smoking‐related morbidities and mortality.

### Inclusion of morbidities

We want decision trees and Markov components to include only those morbidities which are both caused by smoking and are sufficiently prevalent to have meaningful economic impacts. A scoping review identified smoking‐attributable morbidities occurring in pregnancy and in infants 20. Using the criteria outlined above, decision trees include the following maternal morbidities: placental abruption, ectopic pregnancy, pre‐eclampsia, placenta previa and miscarriage (fetal death and expulsion from uterus before 24 weeks). Trees also include the following infant morbidities: low infant birth weight (LBW; i.e. < 2500 g), stillbirth (i.e. born dead after 22 weeks gestation) and premature birth (i.e. born before 37 weeks). For infants in the ‘childhood’ component, we included asthma 21, 22. For both women's and infants’ ‘life‐time’ components, as in other models 17, 23, 24, we included coronary heart disease (CHD) 25, chronic obstructive pulmonary disease (COPD) 26, lung cancer 26 and stroke 27.

### Incorporating maternal smoking behaviour

ESIP incorporates maternal smoking‐related data in four places:

- *In pregnancy—user input*: as above, the proportions of women anticipated to stop smoking in pregnancy both with and without an intervention are inputted and used at the first node in the maternal decision tree (Fig. 1).
- *Maternal ‘life‐time’ component*: in the first two postpartum years, relapse to smoking rates are higher than those generally. For women who were not smoking at childbirth a systematic review indicated their probabilities of returning to smoking within the first and second postpartum years (see Supporting information, Appendix S2) 20, 28. For women who smoked at childbirth, we estimated the percentage who would make a quit attempt in the first postpartum year from the 2010 Infant Feeding Survey (IFS), using data reported at 10 months after childbirth as a proxy for 1‐year data 29.
- *Both ‘life‐time’ components*: except for the situation in (ii) above, we used English ONS smoking data to annually estimate ‘transition probabilities’, the annual probabilities of moving between smoking ‘states’ (see Supporting information, Appendix S2 and section C1 in Appendix S3) 22. To estimate the annual probability of restarting smoking after long abstinence periods, we used 8‐year follow‐up data from a smoking cessation trial (see Supporting information, Appendix S2 and section C2 in Appendix S3) 30.
- *‘Childhood’ component*: we used Health Survey for England data to estimate the proportion of maternal smokers who exposed children to passive smoking 31, and systematic review findings 19 were used to calculate teenagers’ probabilities of starting smoking, given that their mothers smoked (see section C2 in Supporting information, Appendix S3).

### Determining smoking‐related morbidity

To estimate accurately the burden of smoking‐related morbidity, ESIP needed contemporary data on the frequency with which included conditions occurred. For nodes in decision tree components, we sought proportions of women and fetuses developing smoking‐related morbidities. We sourced data on frequency of fetal loss, maternal morbidities and gestational length from Hospital Episode Statistics (HES) NHS Maternity Statistics for England (2006–16) 32 on gestation‐specific infant mortality from Office of National Statistics (ONS), England and Wales (2006–12) 33 and on live births and stillbirths by birth weight and prematurity from Child Mortality Statistics (2006–12) 34. A technical explanation of how the proportions were calculated can be found in section C3 in Supporting information, Appendix S3. To estimate the probabilities that ‘never smokers’ or their fetuses might experience morbidities, we adjusted bootstrapped proportions, using the same approach. Odds ratios and relative risks representing the increased harm caused by smoking during pregnancy came from three systematic reviews 9, 10, 11. Probability estimates can be found in Supporting information, Appendix S2.

For life‐time, childhood and adulthood components, we calculated the number of women/infants with smoking‐related morbidities using English age‐ and gender‐specific prevalence for asthma, CHD, COPD, lung cancer and stroke 35, 36, 37; relative risks for current and former smokers experiencing these were sourced from a US Surgeon General's report (see section C4 in Supporting information, Appendix S3) 38. Where the model predicted that a cohort member had been abstinent for more than 1 year, they were treated as former smokers. For the ‘childhood’ component, we adjusted asthma prevalence for low birth weight and passive smoking exposure, as both may increase asthma risk (see section C5 in Supporting information, Appendix S3) 20, 21, 22, 39, 40. Odds ratios for developing asthma after low birth weight birth and following exposure to maternal smoking in pregnancy and passive smoking came from the literature 40, 41. Prevalence estimates used are shown in Supporting information, Appendix S4.

### Determining smoking‐related mortality

To estimate maternal deaths in pregnancy in ESIP, we used ONS mortality statistics (2006–15) to apply morbidity‐specific probabilities of death with no adjustment for smoking behaviour, as there was no evidence that this influenced chances of dying from within‐pregnancy morbidities (see section C6 in Supporting information, Appendix S3) 42, 43. For the ‘life‐time’ and offspring ‘adulthood’ components, we estimated male and female mortality using ONS cohort life tables 44. Probabilities were adjusted using relative risks from Doll *et al*. to represent the impacts on mortality of smoking and former smoking (see Supporting information, Appendix S4 and section C7 in Appendix S3) 26, 45, with prevalence for current‐, former‐ and never‐smoking taken from ONS data 46. When allocating mortality probabilities, those abstinent for more than 1 year were treated as former smokers. For the ‘childhood’ component, we similarly adjusted mortality rates to allow for the impact of LBW, using odds ratios for LBW‐specific mortality (see section C8 in Supporting information, Appendix S3) 47.

### Determining health‐related quality of life

ESIP calculates the potential for healthy life experience by first awarding ‘life years’ to women and offspring, with the numbers of years lived by cohort members being determined by model components. Life years are then converted into QALYs. We assigned life years to mothers at the end of pregnancy, adjusting for gestational length by assuming that a pregnancy lasts 40 weeks. Informed by HES NHS Maternity Statistics for England, we assumed the average gestational length after ectopic pregnancy was 10 weeks, miscarriage (14 weeks), premature birth (33 weeks), placenta previa (38 weeks) and abruption and pre‐eclampsia (39 weeks) 32. After birth, we first awarded 1 life year to offspring for each year of life.

To generate QALY estimates, life years were weighted by previously estimated utility tariffs 48. We found no evidence to suggest that there was any maternal quality of life loss associated with pre‐eclampsia, and assumed the same for placental abruption and placenta previa 20, 49. A one‐off 0.1 utility loss was applied to all women who experienced a fetal loss (ectopic pregnancy, miscarriage and stillbirth) 50, and was applied in the within‐pregnancy decision tree. For ectopic pregnancy there was an additional one‐off utility loss of 0.01 applied in the within‐pregnancy decision trees 51. Utility weights for smoking‐related morbidities were applied to mothers and offspring aged 16 and above were 0.73 [standard error (SE) = 0.3)] for CHD 52, 0.73 (SE = 0.23) for COPD 53, 0.67 (SE = 0.22) for lung cancer 54 and 0.72 (SE = 0.32) for stroke 55. In the ‘childhood’ component the utility value for offspring aged 1–15 years in perfect health was 1 and, for children with asthma, 0.9 (SE = 0.18) 56.

### Estimating health‐care costs

ESIP's determination of health‐care costs depends on the burden of morbidity estimated. Health‐care events experienced by both mothers and fetuses were counted only once, hence costs were split between maternal and infant components to avoid duplication. Costs attributed only within the maternal components were those of antenatal care, treatment(s) for within‐pregnancy morbidities, stillbirth and mode of delivery. Neonatal care costs for infants born with LBW and prematurity and treatment costs for childhood asthma were attributed only in infant components. Treatment costs associated for ‘life‐time’ morbidities were attributed in both maternal and infant components. ICD‐10 codes were linked to Healthcare Resource Groups (HRG) currency codes, and hence to NHS reference costs (see Supporting information, Appendix S5) 57. Treatment costs for morbidities and delivery were then calculated across the different health‐care settings to estimate a weighted mean 58. The cost of a cardiac event was used as a proxy for the cost of a maternal death 1. A practising NHS midwife advised on attribution of health‐care costs and a detailed explanation of these is shown in Supporting information, Appendix S5. All pregnancies received an antenatal care cost amended for gestational length, morbidity and, for live births, a delivery cost. All live‐born infants received a neonatal care cost based upon length of gestation, using weighted neonatal care costs by the average gestation‐adjusted length of stay in a neonatal intensive care unit 59. Treatment costs for ‘life‐time’ morbidities and childhood asthma were taken from the literature 60, 61, 62, and inflated to 2014–15 prices 63. Individual cost components are shown in Table 1.

**Table 1:** Cost components for ‘within‐pregnancy’, ‘life‐time’, ‘childhood’ and ‘adulthood’ maternal and infant components.

Input	Mean (£)	SE (£)
Within‐pregnancy maternal morbidity treatment		
Ectopic and miscarriage	578.07	226.31
Abruption and previa	1202.38	559.71
Pre‐eclampsia	657.89	329.60
Obstetrician first visit	146.38	68.31
Obstetrician subsequent visit	113.90	62.86
Routine observation after birth	345.24	206.71
Death	1630.98	854.11
Within‐pregnancy maternal birth		
Normal birth	2497.05	745.03
Emergency caesarean section	4180.54	1214.01
Caesarean section	3781.28	1072.94
Stillbirth	1063.28	676.26
Within‐pregnancy maternal ante‐natal care		
Community midwife visit	55.51	17.29
Standard ultrasound scan	110.77	60.65
Specialized ultrasound scan	131.81	50.98
Within‐pregnancy infant delivery		
Neonatal care (premature)	15 934.55	7127.79
Neonatal care (full gestation)	2645.87	2423.44
Childhood treatment		
Asthma	1624.00	162.40
Life‐time morbidity treatment		
CHD	1838.62	183.86
COPD	843.65	84.37
Lung cancer	9554.98	955.50
Stroke	4347.08	29.59

SE = standard error; CHD = coronary heart disease; COPD = chronic obstructive pulmonary disease.

### Incorporating uncertainty

To enable ESIP outputs to reflect the uncertainty of estimates, we fitted distributions enabling probabilistic sensitivity analysis (PSA) using established methods 64. ESIP has 390 variables with fitted distributions and performs 10 000 Monte Carlo simulations to control for uncertainty 65. A technical description can be found in section C9 in Supporting information, Appendix S3.

### Analysis and outcomes

ESIP is constructed in Microsoft Excel 2010 66 and is available online at https://www.nottingham.ac.uk/research/groups/tobaccoandalcohol/smoking‐in‐pregnancy/esip/index.aspx. Because we adopted a UK NHS and PSS perspective 18, costs and benefits accrued after pregnancy (i.e. in life‐time components) were discounted at 3.5% 18. Markov chains are run in annual cycles up to age 100.

The key outcomes ESIP produces are incremental cost‐effectiveness ratios (ICERs) per additional QALY for mother and child, presented both separately and as a combined ‘per pregnancy’ measure of cost‐effectiveness with a ‘life‐time’ perspective. Other outcomes are ICERs per additional life year and per additional quitter, and all outcomes can also be reported to reflect cost‐effectiveness measured only until the end of pregnancy.

Return on investment (ROI) estimates can also be produced for maternal and infant health care, both separately and combined, and reported for all time horizons. These are benefit–cost ratios, defined as incremental health‐care savings divided by incremental intervention cost.

ESIP can also estimate the following ICERs at end of pregnancy: per experience of maternal morbidity, infant death (fetal loss and stillbirth), premature birth, LBW birth and per adverse birth outcomes avoided. Output from the PSA is demonstrated by incremental cost‐effectiveness plane scatterplots and cost‐effectiveness acceptability curves (CEACs), which illustrate the likelihood that an intervention might be judged to be cost‐effective at preselected thresholds.

## Using ESIP with trial data: a worked example

In this section we demonstrate the use of published data from the ‘MiQuit’ pilot randomized controlled trial (RCT) in conjunction with the ESIP model to estimate the cost‐effectiveness of the intervention used: self‐help smoking cessation support delivered by text message 67, 68. Participants were pregnant smokers who received standard NHS smoking cessation care with an intervention group additionally receiving a 12‐week programme of tailored text messages; for full details see Naughton *et al*. 68 No pregnancy outcome data were collected, follow‐up ended at 36 weeks gestation and a simple economic evaluation estimated a ‘cost per quitter’ of £134 [95% confidence interval (CI) = –£396 to £844), basing this only upon intervention costs incurred during pregnancy 68.

### Inputting data

The following data from the trial paper are inputted (values in brackets): mean maternal age (27 years); birth year (2014); per‐participant intervention cost (£3.04, standard error = £0.30) and control and intervention group quit rates (2 and 5.4%). ESIP requires standard errors for cessation outcome data; as these were not reported 67, 68 they were estimated as 1.6 and 1.1%, respectively, using established methods 69, 70. As MiQuit was delivered in addition to standard NHS treatment, we assumed that, in the worst‐case scenario, MiQuit would not improve the chances of a woman quitting over NHS treatment. Therefore, we restricted ESIP to not sample MiQuit quit rates below that of NHS treatment; where this did happen (i.e. sampled MiQuit quit rate was less than sampled NHS treatment), ESIP would assume that the quit rates in the intervention and comparison were equivalent.

### ESIP outputs

Although the published trial economic analysis suggested that the MiQuit intervention was potentially cost‐effective, as measured in ‘cost‐per‐quitter’ units 68, ESIP shows that the greatest health benefits come in the longer term and it also estimates value for money in terms of QALYs, life years and in ROI, none of which was possible in the original trial.

**Figure 3:**
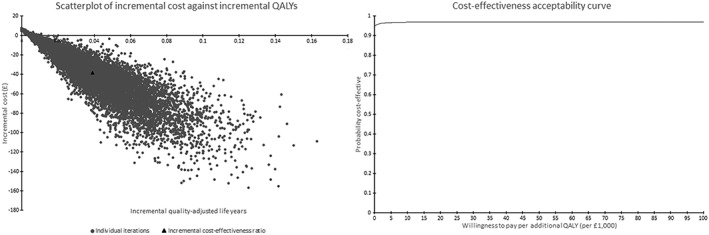
Life‐time horizon probabilistic sensitivity analysis: maternal and offspring costs and outcomes with scatterplot and cost‐effectiveness acceptability curve

More specifically, Table 2 shows base case (using initial model input values with no allowance for uncertainty) and PSA (allows model inputs to vary to estimate the impact of uncertainty) findings. The base case suggests that MiQuit is dominant because it is more effective and cheaper than standard NHS care (incremental cost was negative). The benefit–cost ratio suggests that for every £1 spent on MiQuit the health‐care provider could expect to save £14 per pregnancy throughout the life‐time of the mother and offspring, a finding reinforced by PSA, which demonstrates negative median incremental costs and positive median incremental life years/QALYs with an interquartile range suggesting that the saving could be as little as £8 and as great as £20. The scatterplot of incremental costs versus incremental QALYs (see left‐hand side of Fig. 3) demonstrates that the majority of iterations can be found in the south‐east quadrant (i.e. indicating cost‐effectiveness) 71, and the associated cost‐effectiveness acceptability curve (CEAC) (right‐hand side of Fig. 3) suggests that MiQuit has a probability 0.95 of being cost‐saving, which increases to 0.97 when a decision‐maker is willing to pay £20 000 to gain an additional QALY.

**Table 2:** ESIP MiQuit trial outputs for women and offspring: base case and probabilistic sensitivity analyses, life‐time horizon.

Outcome	Base case (deterministic: no allowance for uncertainty)			Estimates from the probabilistic sensitivity analysis (incorporates uncertainty for model inputs)								
				Comparator			Experimental			Incremental		
	Comparator	Experimental	Incremental	Median	Interquartile range		Median	Interquartile range		Median	Interquartile range	
Maternal outcomes												
Quit rate at delivery (%)	0.0196	0.0542	0.0346	0.0180	0.0119	0.0256	0.0529	0.0431	0.0646	0.0340	0.0214	0.0468
Expected life years per mother	25.1797	25.1827	0.0030	25.2003	25.1634	25.2340	25.2033	25.1670	25.2365	0.0028	0.0018	0.0041
Expected QALYs per mother	23.1165	23.1246	0.0081	23.1946	22.7342	23.5788	23.2028	22.7449	23.5866	0.0074	0.0045	0.0112
Expected cost per mother (£)	10 002.04	9988.28	−13.76	10 033.81	9523.45	10 601.76	10 019.46	9509.49	10 587.53	−13.44	−20.29	−6.99
ICER per additional life year (£)			−4636.23							−4423.80	−5437.41	−3448.47
ICER per additional QALY (£)			−1701.05							−1619.91	−2084.53	−1229.48
ICER per additional quitter (£)			−397.70							−384.84	−461.88	−298.00
Offspring outcomes												
Expected life years per infant	24.0721	24.1020	0.0299	24.0787	23.9804	24.1722	24.1082	24.0143	24.1991	0.0291	0.0181	0.0410
Expected QALYs per infant	23.5449	23.5771	0.0322	23.5953	23.3523	23.7965	23.6282	23.3859	23.8282	0.0310	0.0194	0.0441
Expected cost per infant (£)	7805.18	7777.79	−27.39	7337.74	6409.01	8772.84	7310.38	6380.97	8750.28	−26.37	−39.39	−14.87
ICER per additional life year (£)			−915.58							−884.29	−1104.35	−679.31
ICER per additional QALY (£)			−850.36							−823.64	−1033.68	−625.10
Combined per pregnancy outcomes (mother and offspring)												
Expected life years per pregnancy	49.2519	49.2847	0.0329	49.2754	49.1683	49.3811	49.3091	49.2058	49.4090	0.0321	0.0200	0.0450
Expected QALYs per pregnancy	46.6614	46.7017	0.0403	46.7560	46.1134	47.3147	46.7935	46.1575	47.3575	0.0391	0.0245	0.0552
Expected cost per pregnancy (£)	20 915.76	20 876.48	−39.28	20 677.53	19 251.01	22 428.84	20 638.17	19 212.41	22 383.83	−38.37	−56.96	−21.46
ICER per additional life year (£)			−1194.68							−1150.13	−1422.75	−894.08
ICER per additional QALY (£)			−974.83							−939.53	−1156.12	−737.99
ICER per additional quitter (£)			−1135.27							−1114.32	−1297.95	−909.49
Cost‐offset analysis												
Cost savings ratio for maternal health‐care only (£)			5.53							5.42	3.30	7.78
Cost savings ratio for offspring health‐care only (£)			10.01							9.67	5.84	14.12
Cost savings ratio for combined health‐care only (£)			13.92							13.65	8.02	19.98

ESIP = Economics of Smoking in Pregnancy; QALY = quality‐adjusted life year; ICER = incremental cost‐effectiveness ratio.

Table 3 provides results constrained to the end‐of‐pregnancy time horizon, including estimates for infant morbidities averted by using the intervention. Although ESIP estimates that MiQuit increases maternal QALYs and decreases several adverse infant birth outcomes, it is no longer dominant because it also leads to an increase in cost. However, both base case and PSA ICERs are still below commonly accepted threshold values for QALYs so, even in this analysis, MiQuit appears cost‐effective against standard care as judged by usual norms 72, 73. Benefit–cost ratios suggested that there may be health‐care savings in terms of infant health care; however, this was outweighed by the increased cost associated with the mother.

**Table 3:** ESIP MiQuit trial outputs for women and offspring: base case and probabilistic sensitivity analyses, end‐of‐pregnancy horizon.

Outcome	Base case (deterministic: no allowance for uncertainty)			Estimates from the probabilistic sensitivity analysis (incorporates uncertainty for model inputs)								
				Comparator			Experimental			Incremental		
	Comparator	Experimental	Incremental	Median	Interquartile range		Median	Interquartile range		Median	Interquartile range	
Maternal outcomes												
Number of pregnancies with a morbidity	122	121	−1	122	118	126	121	118	125	−1	−1	0
Expected QALYs per mother	0.6842	0.6852	0.0010	0.6841	0.6779	0.6902	0.6852	0.6790	0.6912	0.0010	0.0006	0.0014
Expected costs per mother (£)	3108.55	3113.45	4.91	3046.26	2618.44	3514.32	3050.83	2622.09	3519.06	4.55	3.72	5.72
ICER per morbidity avoided (£)			6093.90							6385.64	4776.11	9152.32
ICER per additional QALY (£)			4930.28							5251.48	3899.71	7414.55
ICER per additional quitter (£)			141.79							149.78	112.89	206.82
Offspring outcomes												
Number of fetal losses (including stillbirths)	106	105	−1	106	103	110	105	102	109	−1	−1	−1
Number of premature births	73	73	0	73	72	74	73	71	74	0	−1	0
Number of LBW infants	113	111	−2	113	111	115	111	109	113	−2	−3	−1
Total number of infants with adverse birth outcomes	213	211	−2	213	210	217	211	208	215	−2	−3	−1
Expected cost per infant (£)	3261.81	3263.58	1.77	2738.18	1821.11	4154.83	2740.49	1820.83	4156.79	1.94	−0.57	3.66
ICER per adverse birth outcome avoided (£)			892.80							1144.95	−258.57	2973.27
Combined per pregnancy outcomes (mother and offspring)												
Expected cost per pregnancy (mother and infant)	6370.35	6374.00	3.64	5880.56	4832.65	7419.67	5881.73	4834.59	7424.81	3.16	1.24	5.61
ICER per additional QALY (£)			3658.58							4217.09	1235.06	8220.71
ICER per additional quitter (£)			105.22							121.41	34.63	233.44
Cost‐offset analysis												
Cost savings ratio for maternal health‐care only (£)			−0.61							−0.50	−0.88	−0.22
Cost savings ratio for offspring health‐care only (£)			0.42							0.36	−0.19	1.19
Cost savings ratio for combined health‐care only (£)			−1.20							−1.01	−1.83	−0.42

ESIP = Economics of Smoking in Pregnancy; QALY = quality‐adjusted life year; ICER = incremental cost‐effectiveness ratio.

## Discussion

ESIP is the first economic model to acknowledge that maternal smoking in pregnancy and afterwards directly affects fetal and infant pregnancy outcomes, offspring smoking uptake and life‐time experience of smoking‐related illness for both mothers and children. Previous models have considered mothers or infants in isolation 14, 16, 74, 75, and none have incorporated the impact of infants’ exposures to passive smoking. ESIP estimates not only common measures of cost‐effectiveness 13, but also ROI, which may be of interest to decision‐makers.

### Impact of modelling assumptions

By assuming that women who stop smoking in pregnancy have the same risks as those who have never smoked, the model may overestimate the benefits and cost‐effectiveness of cessation in pregnancy. It is probable that ‘quitters’ in pregnancy will have slightly greater risks of experiencing morbidities than ‘never smokers’, as they will have smoked for at least at some of their pregnancies. However, the increase in risk may be small because, for example, when women quit early in pregnancy their infants’ birth weights are no different from those born to non‐smokers 76 and low birth weight is arguably the principal cause of morbidity and mortality among neonates and infants 40, 47. Furthermore, most smokers who stop when pregnant do so early on in pregnancy 77, 78, so this assumption may not affect ESIP outputs greatly.

The model assumes that smoking by household members other than mothers have no impact on either children's passive smoking or their uptake of smoking, so it may underestimate children's smoking‐related morbidity and smoking uptake. If a household has a father or other family members who smoke 19 this has an additional influence on children's smoking uptake, so the benefits attributable to maternal smoking cessation may be overestimated. However, it has been shown that maternal smoking has more impact on children's smoking uptake than paternal smoking 19, 39, so ESIP incorporates the major influence.

The assumption that smoking mothers do not change their smoking behaviour around their child (i.e. either exposing or not exposing them to second‐hand smoking) may mean that ESIP does not estimate children's smoking‐related morbidity accurately. It is unlikely that a mother's smoking behaviour around her child would remain fixed throughout childhood 79, but we could identify no longitudinal data to inform the model about this, and this remains a model limitation. However, the model does attempt to model maternal smoking behaviour after pregnancy accurately, making use of the most recent data on postnatal relapse, and it could be argued that this will have a more substantial impact.

ESIP allows women to make quit attempts after pregnancy, but because Markov models are ‘memoryless’ it assumes each attempt is independent of previous ones 80. However, the more quit attempts an individual makes, the more likely that they are to quit successfully 81, 82, 83, 84, and a woman takes, on average, 6.3 quit attempts throughout her life‐time to become a former smoker 85. ESIP may, therefore, underestimate long‐term abstinence. However, we believe the model makes optimal use of the best available smoking behaviour data and improves on other models by taking into account the different rates of restarting smoking during the first two postnatal years 17, 24.

As incorporating subsequent pregnancies would have been challenging, the model assumes that women have only one pregnancy in their life‐times. The impact this might have on model estimates is uncertain; ESIP may underestimate health‐care costs incurred, but equally it may underestimate siblings’ benefits resulting from mothers’ cessations. In England and Wales in 2013 there was an average of two children in families 86, suggesting that this is a potentially serious limitation. However, if a cessation intervention proved to be cost‐effective for a single child, it seems likely that this would also have benefits for any other children (e.g. in reduced passive smoking) and ESIP estimates would be conservative.

The assumption that MiQuit is superior to standard care removes the possibility of negative incremental quit rates between MiQuit and standard NHS care. The MiQuit pilot study found that there was a non‐significant increase in abstinence [odds ratio (OR) = 2.7, 95% CI = 0.93–9.35] 68, therefore there is the possibility that MiQuit could decrease the likelihood that a woman quits. By not estimating negative incremental quit rates, ESIP could be overestimating the cost‐effectiveness of MiQuit because ESIP is ignoring cases where women and infants are made worse off. However, if MiQuit was to become part of NHS practice in the United Kingdom, it would be delivered in addition to usual care, and hence we considered it additive to usual care rather than replacing it. Furthermore, out of 10 000 replications, this assumption was only applied 345 times, therefore the chance that MiQuit will make women less likely to quit smoking is 3.45%. While this assumption might be made in instances where new interventions are delivered in addition to usual care, it can be relaxed for instances where the new intervention is a direct replacement for usual care, thus ESIP can perform those types of analyses.

The model is restricted to singleton pregnancies. Multiple gestations are infrequent: in 2013 fewer than 2% of all births in England and Wales were multiple births 87; however, pregnancy outcomes in multiple pregnancies are worse than after singleton ones and so health‐care costs are likely to be higher. ESIP would need substantial amendments to account for multiple births; it is not entirely clear how estimates may be affected by excluding the possibility of multiple births but, as these are reasonably rare, any impact is unlikely to be large.

The MiQuit RCT recruited women at an average at 15 weeks gestation and so may have had a limited impact on fetal loss due to miscarriages, which occur principally early in pregnancy; however, we included this outcome in our example, as late miscarriage could be affected by the MiQuit intervention. This may have resulted in some overestimation of MiQuit's cost‐effectiveness in our ‘worked example’, but a positive feature of ESIP is flexibility and it is possible to re‐run analyses removing miscarriage from the list of outcomes as a sensitivity analysis, if desired.

### Application and implication for policy

The ability of ESIP to provide ‘common currency’ outputs (e.g. cost per QALY) is likely to be of most interest to decision‐makers and researchers, as these will allow simple comparisons between cessation interventions delivered in pregnancy and other health‐care interventions. Provided the additional costs of delivering an intervention and the likely (or demonstrated) absolute effect on cessation are known, these can be fed into the programmable interface of ESIP to generate life‐time estimates for intervention cost‐effectiveness without the need for an additional economic model to be built. ESIP inputs currently apply only to the UK population, and so caution is needed when applying ESIP estimates to countries with very different prevalence of smoking behaviours or of smoking‐related illnesses. Model outputs may not be generalizable to such jurisdictions; however, with support, it would be straightforward to re‐parameterize ESIP with other countries’ morbidity and mortality data. ESIP is to be published at: https://www.nottingham.ac.uk/research/groups/tobaccoandalcohol/smoking‐in‐pregnancy/esip/index.aspx and the lead author would able to provide such support.

One consideration with regard to the cost‐effectiveness estimates from the ESIP outputs is to what extent infant outcomes are valued in comparison with maternal outcomes. Currently, there is a lack of international standardization with regard to the inclusion of infant outcomes not only in evaluations of smoking cessation interventions, but also many other pregnancy‐related interventions 88. In the United Kingdom, current guidance on economic evaluations for decision‐making is ambiguous 18. Many previous evaluations of within‐pregnancy smoking cessation interventions have either focused solely on outcomes related to the mother or infant 13; however, several recent interventions have presented a combined measure 17, 24, and thus we presented combined measures of cost‐effectiveness to aid comparison with previous evaluations. It is anticipated that guidance regarding the inclusion of infant outcomes is likely to change, although what this societal decision will be cannot be foreseen. The authors hope that we have demonstrated the flexibility of ESIP in terms of valuing both maternal and infant outcomes, allowing decision‐makers the facility of having the maximum amount of information available to make an informed decision, irrespective of their viewpoint regarding the valuing of infant outcomes.

## Conclusion

ESIP resulted from a systematic approach to address the limitations of previous economic evaluations of smoking cessation interventions used in pregnancy, and offers researchers a comprehensive approach to estimating costs, outcomes and cost‐effectiveness. The inclusion of future cost savings for both mother and child enables decision‐makers to allocate scarce resources with an information set which demonstrates the longer‐term paybacks associated with current investment. Short‐term cost‐effectiveness ratios are misleading when evaluating preventive interventions because future savings are not included, the result being a suboptimal allocation of resources.

## Declaration of interests

None.

## Supporting information


**Appendix S1** Technical summary of methods.

**Appendix S2** Transition probabilities used in model.
**Appendix S3** Prevalence of morbidities and mortality.
**Appendix S4** Detailed figures of model structure.

**Appendix S5** ICD‐10 codes and NHS reference costs codes.
