# Supplementary material for: A dynamic, modifiable model for estimating cost‐effectiveness of smoking cessation interventions in pregnancy: application to an RCT of self‐help delivered by text message
Source: Addiction. 2018 Dec 5;114(2):353–65. doi: 10.1111/add.14476 (PMC6519118; doi:10.1111/add.14476)
Supplement: Supplementary file 2 — Appendix S2 Transition probabilities used in model. Appendix S3 Prevalence of morbidities and mortality. Appendix S4 Detailed figures of model structure. [file ADD-114-353-s002.docx]

**Appendix B: Transition probabilities used in ESIP**

*Table B1: Transition probabilities for smoking behaviour in the maternal ‘lifetime’ and infant ‘adulthood’ components*

| **Input** | **FEMALES** | | **MALES** | | **RR for maternal smoking** | | | **Source (where relevant)** |
| --- | --- | --- | --- | --- | --- | --- | --- | --- |
|  | **Mean** | **SE** | **Mean** | **SE** | **Mean** | **95% CI** | |  |
| **Maternal smoking behaviour up to two years postpartum** | | | | | | | |  |
| Restart smoking within one year of giving birth | .4700 | .0459 | N/A | | | | | Jones et al [1] |
| Restart smoking within two years of giving birth having been abstinent for at least one year after delivery | .2900 | .0561 |  |  |  |  |  | Jones et al [1] |
| Current smoker making a quit attempt during the first year after birth | .1300 | .0130 |  |  |  |  |  | Table 11.20, Infant Feeding Survey [2] |
| **Smoking behaviour outside two years postpartum** | | | | | | | |  |
| Current smoker makes a quit attempt | .2700 | .0339 | 0.250 | .0250 | N/A | | | Table 3.6, Statistics on Smoking: England [3] |
| Quitter remains abstinent for more than one year but less than two | .1400 | .0140 | Same as females | |  |  |  | Table 3.7, Statistics on Smoking: England [3] |
| Quitter remains abstinent for longer than two years (becomes long term quitter) | .5714* | .0823 |  |  |  |  |  | See note below |
| Long term quitter restarts smoking | .0086† | .0009 |  |  |  |  |  | Yudkin et al [4] |
| **Exposure to passive smoking** | | | | | | | | |
| Probability that an actively smoking mother (either current or restarting) will expose their offspring to passive smoking | .4775 | .1515 | N/A | | | | | Analysis of Health Survey for England data[5] |
| **Probability of picking up smoking conditional on smoking behaviour** | | | | | | | |  |
| Start smoking given a non-smoking mother, aged under 16 years | .1153 | .0065 | .1881 | .0080 | 1.86 | 1.54 | 2.23 | See section A1 in Appendix A for how these values were estimated |
| Start smoking given a non-smoking mother, aged 16-17 years | .0519 | .0033 | .0803 | .0041 | 2.04 | 1.64 | 2.52 |  |
| Start smoking given a non-smoking mother, aged 18-19 years | .0409 | .0029 | .0656 | .0039 | 2.07 | 1.65 | 2.57 |  |
| Start smoking given a non-smoking mother, aged 20-24 years | .0157 | .0011 | .0265 | .0016 | 1 | | |  |
| Start smoking given a non-smoking mother, aged 25 years and over | .0019 | .0001 | .0015 | .0001 |  |  |  |  |
| *=Cross sectional data [3] estimated that 14% of quitters had been abstinent for less than one year, but 8% had been abstinent for more than two years, hence probability defined as 8/14 = 0.57 | | | | | | | | |
| †= Yudkin et al estimated that at one year 153 individuals were abstinent but by eight years this had reduced to 83. [4] Using .5714 to estimate how many were abstinent at 2 years (87), we estimated that four restarted smoking between two and eight years, and this was divided by 87 and converted into an annual transition probability using the method by Fleurence et al. [6] | | | | | | | | |

*Table B2: Estimated probabilities of within-pregnancy outcomes for mother and infants with associated standard error and RRs for smoking as used in ESIP; for details of how these values were generated see section Agh in Appendix A*

| **Input** | **Probability amongst never smokers** | | **RR for smoking** | | |
| --- | --- | --- | --- | --- | --- |
|  | **Mean** | **Standard error** | **Mean** | **95% CI** | |
| **Early stage foetal loss before 24 weeks gestation** | | | | | |
| Ectopic pregnancy | .0139 | .0004 | 1.77 | 1.37 | 2.26 |
| Miscarriage | .0572 | .0011 | 1.30 | 1.16 | 1.44 |
| No foetal loss | .9290 | .0011 | N/A | | |
| **Maternal morbidity 24 weeks gestation and beyond** | | | | | |
| Placenta abruption | .0036 | .0001 | 1.62 | 1.47 | 1.78 |
| Placenta previa | .0060 | .0002 | 1.60 | 1.10 | 2.23 |
| Pre-eclampsia | .0203 | .0003 | 0.52 | 0.40 | 0.67 |
| No morbidity | .9700 | .0004 | N/A | | |
| **Premature birth given maternal morbidity** | | | | | |
| Placenta abruption | .3775 | .0057 | 1.25 | 1.20 | 1.31 |
| Placenta previa | .2529 | .0022 |  |  |  |
| Pre-eclampsia | .2620 | .0029 |  |  |  |
| No morbidity | .0591 | .0010 |  |  |  |
| **LBW conditional on gestation** | | | | | |
| Premature | .5704 | .0056 | 1.82* | 1.67* | 1.97* |
| Full gestation | .0269 | .0003 |  |  |  |
| **Stillbirth (foetal loss at 24 weeks and beyond) conditional on gestation and birthweight** | | | | | |
| LBW and premature | .0608 | .0006 | 1.26* | 1.19* | 1.34* |
| NBW and premature | .0076 | .0005 |  |  |  |
| LBW and full gestation | .0154 | .0012 |  |  |  |
| NBW and full gestation | .0016 | .0000 |  |  |  |
| **Maternal mortality conditional on morbidity** | | | | | |
| Ectopic pregnancy | .0001 | .0000 | N/A | | |
| Miscarriage | .0000 | .0000 |  |  |  |
| Placenta abruption | .0003 | .0001 |  |  |  |
| Placenta previa | .0001 | .0000 |  |  |  |
| Pre-eclampsia | .0002 | .0000 |  |  |  |
| No morbidity | .0001 | .0000 |  |  |  |
| *=Not estimated from the bootstrap process, source: DiFranza et al [7] | | | | | |

**Appendix C: Technical Summary of Methods.**

*C1: Incorporating maternal smoking behaviour*

We used ONS cross-sectional data on smoking behaviour in England to estimate transition probabilities to represent changes in the mothers smoking behaviour. [3] The probabilities estimated can be found in Table 1 in Appendix B. From ONS data tables [3], it was found that amongst smokers in 2008/09 who had made a previous quit attempt, 6% had reported being abstinent for at least one year but had restarted smoking before two years, and 8% reported being abstinent for more than two years. To estimate the probability of a quitter maintain a quit attempt for at least one year, we summed these two percentages together to give 14% (suggesting 86% return to smoking within one year of starting a quit attempt). We used this value to estimate the proportion of quitters who transited from the ‘0-1 year quitter’ state to the ‘1-2 year quitter’ state (see the lifetime Markov diagrams in Appendix A). To estimate the transition probability for a quitter becoming a long term quitter (i.e. abstinent for more than two years since start of quit attempt), we simply divided the 8% by 14% to give 0.57 approximately (hence 43% appear to return to smoking). We used this value to estimate the proportion of quitters who transited from the ‘1-2 year quitter’ state to the ‘long term quitter’ state (see the lifetime Markov diagrams in Appendix A). To take into account that ‘long term quitters’ can still return to smoking, we estimated a transition probability of returning to smoking beyond two years using data from a smoking cessation RCT with an 8 year follow up period. [4] Yudkin et al estimated that at one year follow-up 153 individuals were abstinent but by eight years follow up 70 individuals had relapsed. Allowing for individuals to return to smoking between one and two years using our value above, we estimated that 87 participants were abstinent at year two, and hence four participants restarted smoking between two and eight years after a quit attempt. This estimated a probability of 0.0507, which was then converted into an annual transition probability using the method by Fleurence et al. [6] This annual transition probability was then applied in all cycles of the model.

*C2: Estimating ESIP inputs for the probabilities of picking up smoking amongst offspring controlling for their mother’s smoking*

From 15 years of age onwards, the offspring ‘adulthood’ component introduces into the model young people’s smoking uptake, influenced by maternal smoking. For ESIP to accurately estimate this behaviour, we required to not only to identify estimates for the influence of maternal smoking, but also how the offspring transit being never smokers to current smokers.

The starting point was to identify at what age most individuals will have started smoking. We did this by identifying the age at which fewest respondents reported having never smoked in Office of National Statistics (ONS) cross sectional data from across Britain. [8] The ONS data reported that for men the lowest proportion of never smokers was age 60 years (proportion never smoked, 0.4503) and for women the corresponding age was 49 years (proportion, 0.6058). By subtracting these values from one we were able to calculate the proportion that were ‘ever smokers’.

However, most individuals start smoking at a younger age, and so to reflect this we required to weight the proportion of ever smokers. We did this by using Health Survey for England data [5] (see Table C2.1 below), for example, we estimated the probability that males who started smoking before age 16 irrespective of their mother’s smoking status as 0.2184 by simply multiplying the proportion of ‘ever smokers’ (0.5497) by the proportion who started smoking aged 15 years and under (0.3973). Similarly, for ages 16-17 years and older, we calculated the probability that a male would start smoking between 16 and 17 years irrespective of mother’s smoking status as 0.1814, by first repeating the above process to estimate the proportion of ‘ever smokers’ who started smoking aged 16-17 years ($0.5497\times0.2580=0.1418$), and then dividing this by the proportion who were ‘never smokers’ when aged 15 years or less ($0.1418\div\left( 1-0.2184 \right)=.1814$). The process was repeated for higher age categories; proportions were converted to annual transition probabilities using established methods. [6]

Table C2.1: Age at which an individual started smoking

| **Age range (years** | **Males** | | | **Females** | | |
| --- | --- | --- | --- | --- | --- | --- |
|  | **Mean proportion** | **95% confidence interval (CI)** | | **Mean proportion** | **95% CI** | |
| **0-15** | 0.3973 | 0.3972 | 0.3974 | 0.3446 | 0.3445 | 0.3447 |
| **16-17** | 0.2580 | 0.2580 | 0.2580 | 0.2629 | 0.2629 | 0.2630 |
| **18-19** | 0.1744 | 0.1744 | 0.1745 | 0.1840 | 0.1839 | 0.1841 |
| **20-24** | 0.1243 | 0.1242 | 0.1244 | 0.1326 | 0.1325 | 0.1327 |
| **25+** | 0.0461 | 0.0460 | 0.0461 | 0.0759 | 0.0758 | 0.0759 |
| ***Source:*** | *Analysis of Health Survey for England data, 2006-2014 [5]* | | | | | |

To incorporate the impact of maternal smoking on young people’s smoking uptake, we adjusted above values using the Flack’s method. [9] To calculate the probability of an occurrence amongst never smokers (or in this case infants where their mother is a non-smoker), we used the following equation:

$Probability\left( Event | Never smoker \right)= \frac{Probability\left( Event | Population \right)}{{Prevalence}_{Never smoking}+\left( {Prevalence}_{Smoking}\times RR \right)}$ (1)

Where RR is the relative risk of the increased chance an event will occur attributable to smoking. However, the measure of the impact of maternal smoking has on the likelihood that their offspring becomes a smoker up to age 19 years was the odds ratio of 2.19 (95% confidence interval (CI) 1.73 to 2.79).[10] To apply this odds ratio required converting the probability into an odds using the following formula:

$Odds\left( Event \right)=\frac{Probability(Event)}{1-Probility(Event)}$ (2)

Continuing the example from above, the probability that a male was smoking before age 16 was 0.2184 irrespective of their mother’s smoking status, which is 0.2794 once converted in an odds using equation 2. We can now apply the odds ratio instead of the relative risk in equation 1 by simply replacing the probability (Event|Population) with the odds (Event|Population, thus calculating the odds (Event|Population):

$Odds\left( Offspring starts smoking | Mother is not smoking \right)= \frac{0.2794}{{Prevalence}_{Mother not smoking}+({Prevalence}_{Mother smoking}\times2.19)}$ (3)

To inform the prevalence of maternal smoking / never smoking, we used 0.1723 (the proportion of all women aged 16 years or over who reported cigarette smoking in 2014) as a proxy for the prevalence of women who ever smoke in the UK [8] (and hence the prevalence of mothers not smoking is one minus the prevalence of mothers smoking), the denominator becomes:

$$Odds\left( Offspring starts smoking | Mother is not smoking \right)=$$

$\frac{0.2794}{(1-0.1723)+(0.1723\times2.19)}=0.2319$ (4)

The odds that the offspring will start smoking given the mother is a smoker is simply the multiplication of the odds ratio by the odds of the offspring starting smoking given the mother is not a smoker:

$Odds\left( Offspring starts smoking | Mother is a smoker \right)=2.19\times0.2319=0.5079$ (5)

To convert the odds back to probabilities requires the reverse of equation 2:

$Probability\left( Event \right)=\frac{Odds(Event)}{1+Odds(Event)}$ (6)

Continuing our example, the probability that a male offspring will start smoking by age 16 given the mother is not smoking is $\frac{0.2319}{1+0.2319}=0.1882$, while the probability that a male offspring will start smoking by age 16 given their mother is a smoker is $\frac{0.5079}{1+0.5079}=0.3368$. This was repeated for all age categories and for females.

To control for uncertainty, we bootstrapped these estimates 10,000 times to calculate appropriate distributions for the model parameters. [11, 12] Additionally, to aid model simplicity we calculated relative risks based on the bootstrap results instead of using the odds ratios. This was simply calculated by dividing the probability that the offspring would start smoking given the mother was a smoker by the probability the offspring would start smoking given the mother was a non-smoker. After 20 years of age, we assumed maternal smoking had no influence smoking uptake and did not adjust for this. The values that this process estimated and used for ESIP inputs can be found in Appendix B, Table B2.

*C3: Estimating probabilities for maternal and infant morbidities within pregnancy conditional on smoking behaviour*

For ESIP to estimate the impact of smoking / smoking cessation interventions have on the maternal and infant experience during pregnancy, we were required to identify suitable probabilities for the occurrence of each morbidity during pregnancy. Frequency data (by year) on ectopic pregnancy, miscarriage, placental abruption, placenta previa, pre-eclampsia, and number of delivery episodes was sought from Hospital Episode Statistics (HES) NHS Maternity Statistics for England (2006-2016). [13] This was done for each year between 2006 and 2016. The frequency of each morbidity was then divided by the number of delivery episodes, except for Ectopic pregnancy and Miscarriage which was divided by the number of conceptions (defined as number of births plus ectopic pregnancy events plus miscarriage events) to calculate a probability of morbidity irrespective of smoking status of the mother. For estimating the probabilities that an infant would be born prematurely with respect to each morbidity, frequency data was again sought from HES NHS Maternity Statistics for England (2012-2016) [13], however this was only reported for 2012 onwards; the frequency of premature births amongst mothers suffering / not suffering a condition was divided by the number of pregnancies affected by each condition respectively to give a probability of premature birth irrespective of maternal smoking status. To estimate the number of infants born with low birth weight and / or stillborn, frequency data was sought from Office of National Statistics (ONS) Gestation-specific infant mortality in England and Wales (2006 to 2012) [14] for the number of live births by gestation – (premature vs full), while Child Mortality Statistics (2006 to 2012) [15-17] gave numbers of stillbirths by birthweight (low birth weight vs normal birth weight) and gestation length (premature vs full). To calculate the probability that a premature infant was born with low birth weight, we divided the number of premature infants born with low birth weight by the number of premature of births reported in the ONS datasets. This was repeated for full gestation infants, given two probabilities of an infant being born with low birth weight irrespective of smoking behaviour. For the probability of stillbirth amongst premature low birth weight infants, the number of stillbirths amongst premature low birth weight infants were divided by the number of infants born prematurely with low birth weight in the ONS datasets. This was repeated for stillbirth amongst premature normal birth weight infants, stillbirth amongst full gestation low birth weight infants, and stillbirth amongst full gestation normal birthweight infants to give a probability of stillbirth controlling for birth weight and gestation irrespective of maternal smoking status during pregnancy.

Probabilities of each morbidity were calculated on a per annum basis. To enable ESIP to include the variability associated with these probabilities, we bootstrapped with replacement the annual probabilities of each morbidity 10,000 times using Stata [18]. To capture the impact of maternal smoking during pregnancy on the risk of each morbidity, we identified relevant odds ratios / relative risks for each condition quantifying the impact of smoking. These can be found in Table C3.1.

Table C3.1: Identified odds ratios and relative risks for within-pregnancy conditions quantifying the impact of maternal smoking during pregnancy

| **Morbidity** | **Mean estimate** | **95% confidence interval (CI)** | | **Source** |
| --- | --- | --- | --- | --- |
| ***Odds ratios*** |  |  |  |  |
| **Ectopic pregnancy** | 1.77 | 1.31 | 2.22 | Castles et al [19] |
| **Miscarriage** | 1.32 | 1.18 | 1.48 | DiFranza et al [7] |
| **Placental abruption** | 1.62 | 1.46 | 1.77 | Castles et al [19] |
| **Placenta previa** | 1.58 | 1.04 | 2.12 | Castles et al [19] |
| **Pre-eclampsia** | 0.51 | 0.38 | 0.64 | Castles et al [19] |
| **Premature birth** | 1.27 | 1.21 | 1.33 | Shah et al [20] |
| ***Relative risks*** |  |  |  |  |
| **Low birth weight** | 1.82 | 1.67 | 1.97 | DiFranza et al [7] |
| **Stillbirth** | 1.26 | 1.19 | 1.34 | DiFranza et al [7] |

Each odds ratio / relative risk was bootstrapped 10,000 times using the log normal distribution. [21] To apply the relative risks, we used the same approach as in Equation 1, whereby the probability of the condition irrespective of smoking behaviour is divided by the sum of the prevalence of maternal not smoking at birth and the prevalence of maternal smoking at birth by the relative risk associated with maternal smoking. We used ONS estimates for the prevalence of maternal smoking at birth (11.38%, 95% CI 11.30%-11.46%). [22] This was done for each of our 10,000 sampled probabilities and odds ratios, giving us the probability of each condition amongst never smokers (see Table B2 in Appendix B for estimated values). The probability of the condition amongst smokers could thusly be calculated by multiplying the never smoker probability by the relative risk associated with maternal smoking in pregnancy:

$P\left( Morbidity | Smoking in pregnancy \right)=P(Morbidity|Not smoking in pregnancy)\times{RR}_{SIP}$ (7)

Where ${RR}_{SIP}$ is the relative risk of maternal smoking in pregnancy. For the odds ratios, we first had to convert the probability to an odds using equation 2 above. The odds of the morbidity amongst never smokers can then be calculated thusly:

$Odds\left( Morbidity \right|Not smoking in pregnancy)= \frac{Odds (Morbidity)}{\left( {Prevalence}_{SIB}\times{OR}_{SIP} \right)+{Prevalence}_{NSIB}}$ (8)

Where ${Prevalence}_{SIB}$ is the prevalence of smoking at birth, ${Prevalence}_{NSIB}$ is the prevalence of not smoking at birth (and hence is one minus ${Prevalence}_{SIB}$), and ${OR}_{SIP}$ is the odds ratio for smoking during pregnancy. The odds of the morbidity amongst those who smoke during pregnancy can then be calculated thusly:

$$Odds\left( Morbidity | Smoking in pregnancy \right)=$$

$Odds\left( Morbidity \right|Not smoking in pregnancy) \times{OR}_{SIP}$ (9)

The probability of the morbidity amongst those smoking / not smoking during pregnancy can then be calculated by converting the odds using equation 6 above. This was done for each of the 10,000 sampled probabilities and odds ratios / relative risks, giving a mean probability of morbidity amongst those not smoking in pregnancy and associated standard error which were used for ESIP (see Table B2 in Appendix B). For modelling simplicity, rather than use the odds ratios, we calculated the relative risk of each morbidity (calculated by dividing the probability of the morbidity amongst smokers by the probability of the morbidity amongst those not smoking in pregnancy) for each of the bootstrapped replications, giving a mean relative risk and 95% confidence interval (see Table B2 in Appendix B for our estimated values).

To demonstrate these calculations in practice, we will work through a simplified calculation for estimating the probability of ectopic pregnancy conditional on within-pregnancy smoking behaviour. From HES data [13], in 2015, 10,542 women suffered from Ectopic pregnancy while there 696,998 conceptions. The probability that a women irrespective of smoking status will suffer from an ectopic pregnancy is therefore:

$Probability\left( Ectopic pregnancy \right)= \frac{10,542}{696,998}=0.0151$ (10)

Using equation 2, the odds of ectopic pregnancy irrespective of smoking status can be estimated as:

$Odds \left( Ectopic pregnancy \right)=\frac{0.0151}{1-0.0151}=0.0153$ (11)

From Table C3.1, the odds ratio that a conception will result in an ectopic pregnancy given the mother is a smoker is 1.77 [19], while an estimated 11.38% of women are still smoking at delivery. [22] By substituting these values into equation 8, the odds that a conception will result in an ectopic pregnancy for a never smoker is:

$Odds\left( Ectopic pregnancy | Not smoking in pregnancy \right)=\frac{0.0153}{0.8862+(0.1138\times1.77)}=0.0141$ (12)

The odds that a conception will result in an ectopic pregnancy given the mother is estimated by using the odds ratio and the above value in equation 9:

$Odds\left( Ectopic pregnancy \right|Smoking in pregnancy)=0.0141\times1.77=0.0250$ (13)

Finally the probabilities for ectopic pregnancy conditional on smoking behaviour can be calculated by converting the two estimated odds using equation 6:

$Probability\left( Ectopic pregnancy | Not smoking in pregnancy \right)=\frac{0.0141}{1+0.0141}=0.0139$ (14)

$Probability\left( Ectopic pregnancy | Smoking in pregnancy \right)=\frac{0.0250}{1+0.0250}=0.0244$ (15)

*C4: Estimating ESIP inputs for chronic morbidities in the ‘lifetime’ components of ESIP*

For ESIP to estimate the prevalence of chronic morbidities (CHD, COPD, LC, and Stroke) in the maternal ‘lifetime’ and infant ‘adulthood’ components, we required suitable inputs that would represent the prevalence of each morbidity amongst never smokers which could then be adjusted for smoking behaviour using a suitable relative risk. This we required the calculation of the prevalence amongst never smokers for each morbidity. To do this, we used the same approach as described above in equation 1, but adjusting the equation to include ‘former smokers’, as per Flack et al. [9]

$Prevalence\left( {Morbidity}_{T} | {Never smoker}_{T} \right)= \frac{Prevalence({Morbidity}_{P,T})}{{Prevalence}_{NS, T}+\left( {Prevalence}_{CS,T}\times{RR}_{CS ,T} \right)+({Prevalence}_{FS, T}\times{RR}_{FS, T})}$ (16)

Where $\left( {Morbidity}_{T} | {Never smoker}_{T} \right)$ represents the prevalence of each disease amongst never smokers at a given age T, ${Morbidity}_{P,T}$ represents the prevalence of each disease amongst the general population regardless of smoking behaviour at age T, ${Prevalence}_{NS, T}$ represents the prevalence of never smokers at age T,${Prevalence}_{CS,T}$ represents the prevalence of current smokers at age T, ${Prevalence}_{FS, T}$ represents the prevalence of former smokers at age T, ${RR}_{CS ,T}$ is the relative risk for the disease amongst those who are current smokers at age T, and ${RR}_{FS, T}$ is the relative risk for the disease amongst former smokers at age T.

To calculate the required model input, the first required value is the prevalence of the morbidity at given age irrespective of the smoking behaviour of individuals. This information can be found in Table C4.1.

*Table C4.1: Prevalence of morbidities amongst the general population irrespective of smoking behaviour used to estimate ESIP morbidity inputs*

|  | **Prevalence of condition (%)** | | | | | | | |
| --- | --- | --- | --- | --- | --- | --- | --- | --- |
| **Age range(years)** | **Coronary Heart Disease (CHD)** | | **Chronic Obstructive Pulmonary Disorder (COPD)** | | **Lung Cancer (LC)** | | **Stroke** | |
|  | **Males** | **Females** | **Males** | **Females** | **Males** | **Females** | **Males** | **Females** |
| 0-4 | 0.0 | 0.0 | 0.0 | 0.0 | 0.0 | 0.0 | 0.0 | 0.0 |
| 5-9 | 0.0 | 0.0 | 0.0 | 0.0 | 0.0 | 0.0 | 0.0 | 0.0 |
| 10-14 | 0.0 | 0.0 | 0.0 | 0.0 | 0.0 | 0.0 | 0.0 | 0.0 |
| 15-19 | 0.1 | 0.1 | 0.0 | 0.0 | 0.0 | 0.0 | 0.0 | 0.2 |
| 20-24 | 0.1 | 0.1 | 0.0 | 0.0 | 0.0 | 0.0 | 0.0 | 0.2 |
| 25-29 | 0.2 | 0.1 | 0.0 | 0.0 | 0.0 | 0.0 | 0.0 | 0.1 |
| 30-34 | 0.2 | 0.1 | 0.1 | 0.0 | 0.0 | 0.0 | 0.0 | 0.1 |
| 35-39 | 0.6 | 0.3 | 0.1 | 0.1 | 0.0 | 0.0 | 0.5 | 0.4 |
| 40-44 | 0.6 | 0.3 | 0.2 | 0.2 | 0.0 | 0.0 | 0.5 | 0.4 |
| 45-49 | 3.6 | 1.3 | 0.5 | 0.5 | 0.2 | 0.1 | 1.2 | 0.9 |
| 50-54 | 3.6 | 1.3 | 1.0 | 1.1 | 0.2 | 0.1 | 1.2 | 0.9 |
| 55-59 | 10.6 | 3.5 | 2.0 | 2.0 | 0.2 | 0.1 | 3.0 | 2.3 |
| 60-64 | 10.6 | 3.5 | 3.5 | 3.1 | 0.2 | 0.1 | 3.0 | 2.3 |
| 65-69 | 20.8 | 10.0 | 5.8 | 4.7 | 0.8 | 0.2 | 7.1 | 4.2 |
| 70-74 | 20.8 | 10.0 | 7.9 | 5.7 | 0.8 | 0.2 | 7.1 | 4.2 |
| 75-79 | 28.6 | 19.3 | 9.4 | 6.8 | 0.8 | 0.2 | 13.1 | 10.7 |
| 80-84 | 28.6 | 19.3 | 10.6 | 6.8 | 0.8 | 0.2 | 13.1 | 10.7 |
| 85+ | 28.6 | 19.3 | 9.9 | 5.3 | 0.8 | 0.2 | 13.1 | 10.7 |
| *Source:* | *Table 2.13, Compendium of CHD Statistics 2012 [23]* | | *Flowers et al, Public Health England Dataset [24]* | | *Table 3, Forman et al, EUROPEVAL study [25]* | | *Table 2.13, Compendium of CHD Statistics 2012 [23]* | |

The second required value is the prevalence of each of the different smoking behaviours (never, current, and former smoker) at a given age. This information can be found in Table C4.2.

*Table C4.2: Prevalence of current, former, and never smokers in Great Britain, by age and sex, 2014*

| **Age range (years)** | **Prevalence of smoking behaviour amongst males (%)** | | | | | | | **Prevalence of smoking behaviour amongst females (%)** | | | | | | |
| --- | --- | --- | --- | --- | --- | --- | --- | --- | --- | --- | --- | --- | --- | --- |
|  | **Current Smokers** | | | **Never smokers** | | | **Former smokers†** | **Current Smokers** | | | **Never smokers** | | | **Former smokers†** |
|  | **Mean** | **95% CI** | | **Mean** | **95% CI** | |  | **Mean** | **95% CI** | | **Mean** | **95% CI** | |  |
| **0-15*** | 0.00 | N/A | | 100.00 | N/A | | 0.00 | 0.00 | N/A | | 100.00 | N/A | | 0.00 |
| **16-24** | 25.21 | 20.09 | 30.34 | 69.71 | 64.43 | 74.99 | 5.07 | 20.86 | 16.60 | 25.12 | 72.63 | 67.74 | 77.52 | 6.51 |
| **25-34** | 26.49 | 22.50 | 30.48 | 55.98 | 51.63 | 60.34 | 17.52 | 21.84 | 18.70 | 24.99 | 61.60 | 57.65 | 65.56 | 16.55 |
| **35-49** | 22.45 | 19.91 | 25.00 | 54.28 | 51.09 | 57.48 | 23.26 | 19.61 | 17.16 | 22.05 | 60.58 | 57.37 | 63.78 | 19.82 |
| **50-59** | 19.77 | 16.49 | 23.06 | 55.49 | 51.37 | 59.61 | 24.74 | 18.05 | 15.02 | 21.08 | 61.26 | 57.27 | 65.26 | 20.69 |
| **60+** | 12.40 | 10.57 | 14.23 | 45.03 | 42.30 | 47.77 | 42.57 | 10.62 | 90.80 | 12.16 | 61.05 | 58.51 | 63.59 | 28.33 |
| **Source:** | Table 14, Adult Smoking Habits in Britain, 2014 [8] | | | | | | | | | | | | | |
| * = ONS estimates do not include anyone under the age of 16 years, and therefore for modelling simplicity it is assumed that everyone is a never smoker between the ages of 0 and 15 years | | | | | | | | | | | | | | |
| † = ONS estimates do not include the prevalence of former smokers, however it can be worked out by subtracting the sum of current and never smokers from 100%, as done here for mean values only as an example | | | | | | | | | | | | | | |

The final information required is the associated relative risk for each morbidity associated with current and former smokers (never smokers are assumed to have a relative risk equal to one). This information can be found in Table C4.3.

*Table C4.3: Relative risks for each morbidity associated with smoking behaviour (current and former), by age and gender*

| **Age range (years)** | **Current smokers** | | | | | | | | **Former smokers** | | | | | | | |
| --- | --- | --- | --- | --- | --- | --- | --- | --- | --- | --- | --- | --- | --- | --- | --- | --- |
|  | **Males** | | | | **Females** | | | | **Males** | | | | **Females** | | | |
|  | **CHD** | **COPD** | **LC** | **Stroke** | **CHD** | **COPD** | **LC** | **Stroke** | **CHD** | **COPD** | **LC** | **Stroke** | **CHD** | **COPD** | **LC** | **Stroke** |
| **0-34** | 1 | 1 | 1 | 1 | 1 | 1 | 1 | 1 | 1 | 1 | 1 | 1 | 1 | 1 | 1 | 1 |
| **35-54** | 3.88 | 4.47 | 14.33 | 2.4 | 4.98 | 6.43 | 13.3 | 2.44 | 1.83 | 2.22 | 4.4 | 1.07 | 2.23 | 1.85 | 2.64 | 1 |
| **55-64** | 2.99 | 15.17 | 19.03 | 2.51 | 3.25 | 9 | 18.95 | 1.98 | 1.52 | 3.98 | 4.57 | 1.51 | 1.21 | 4.84 | 5 | 1.1 |
| **65-74** | 2.76 | 29.69 | 28.29 | 2.17 | 3.29 | 38.89 | 23.65 | 2.27 | 1.58 | 8.13 | 7.79 | 1.23 | 1.56 | 15.72 | 6.8 | 1.24 |
| **75+** | 1.98 | 23.01 | 22.51 | 1.48 | 2.25 | 20.96 | 23.08 | 1.7 | 1.32 | 6.55 | 6.46 | 1.12 | 1.42 | 7.06 | 6.38 | 1.1 |
| **Source:** | Table 12.3 US Surgeon General Report [26] | | | | | | | | | | | | | | | |

The prevalence amongst never smokers by age and gender was estimated for each condition using equation 6. These values were then bootstrapped 10,000 times to take into account variability reported in the data to generate mean prevalence amongst never smokers and associated standard errors. The ESIP inputs generated by this process can be found in Appendix D, Table 1.

*C5: Estimating ESIP inputs for asthma in the ‘childhood’ component taking into account maternal smoking behaviour both within and after pregnancy, and birth weight.*

For ESIP to estimate the prevalence of childhood asthma in the infant ‘childhood’ component, we required suitable model inputs. However, it was complicated by the need to not only take into account passive smoking, but also the birth weight of the child [27], and whether they had been exposed to smoking while as a foetus (i.e. the mother smoked during pregnancy). [28] We decided that exposure to passive smoking was the influence of most interest once an infant had entered into the ‘childhood’ component, and therefore decided to control for the other co-morbidities (birth weight and within-pregnancy smoking behaviour) by calculating four prevalence’s where the child wasn’t passive smoking:

*Prevalence (Asthma| Not passive smoking| Mother doesn’t smoke in pregnancy| Normal birth weight)*

*Prevalence (Asthma| Not passive smoking | Mother smokes in pregnancy| Normal birth weight)*

*Prevalence (Asthma| Not passive smoking | Mother doesn’t smoke in pregnancy| Low birth weight)*

*Prevalence (Asthma| Not passive smoking | Mother smokes in pregnancy| Low birth weight)*

To estimate these probabilities, we started by estimating the prevalence of asthma amongst those who were normal birth weight by using the following equation:

$Prevalence (Asthma|NBW)= \frac{Prevalence({Asthma}_{GP})}{(Prevalence(LBW)\times OR)+Prevalence (NBW)}$ (17)

Where $Prevalence ({Asthma}_{GP})$ is the prevalence of asthma amongst the general population irrespective of birth weight and maternal smoking behaviour, $Prevalence (LBW)$is the prevalence of low birth weight (<2500 grams) infants at birth, $Prevalence (NBW)$ is the prevalence of normal birth weight (≥2500 grams) infants at birth (i.e. one minus $Prevalence(LBW)$, and ${OR}_{LBW}$ is the odds ratio of asthma given an infant is born with LBW. The prevalence of asthma amongst the general population irrespective of birth weight and maternal smoking behaviour can be found in Table C5.1. We used the odds ratio from Mu et al for the impact of low birth weight (1.28, 95% CI 1.09-1.5) [27], and estimated that the prevalence of infants born with low birth weight to be 7.4337% (95% CI 7.3015%-7.5660%) from ONS data we had used in section xyz above. [29]

Table C5.1: Prevalence of asthma by age and gender amongst the general population irrespective of birth weight and maternal smoking status

| **Age range (years)** | **Prevalence of asthma (%)** | |
| --- | --- | --- |
|  | **Males** | **Females** |
| **0-4** | 2.29% | 1.36% |
| **5-9** | 11.41% | 7.88% |
| **10-14** | 19.18% | 14.11% |
| **15-19** | 22.34% | 18.27% |
| ***Source:*** | *Flowers et al, Public Health England Dataset [24]* | |

Once we had estimated the prevalence of asthma amongst those born to normal birth weight and low birth weight respectively, we then adjusted for maternal smoking behaviour within pregnancy, using the following formula:

$Prevalence \left( Asthma \right|Mother doesn^{'}t smoke in pregnancy |NBW)= \frac{Prevalence (Asthma|NBW)}{(Prevalence(SAB)\times{OR}_{SAB})+Prevalence (NSAB)}$ (18)

Where $Prevalence (Asthma|NBW)$ is the prevalence of asthma amongst infants born with normal birthweight irrespective of maternal smoking behaviour, $Prevalence(SAB)$ is the prevalence of maternal smoking at birth, $Prevalence (NSAB)$ is the prevalence of maternal non-smoking at birth (and hence is one minus $Prevalence(SAB))$, and ${OR}_{SAB}$ is the odds ratio of asthma given the mother was a smoker at birth. We used ONS estimates for the prevalence of maternal smoking at birth (11.38%, 95% CI 11.30%-11.46%). [22] The odds ratios for the impact of maternal smoking can be found in Table C5.2. This process was also repeated to give values for the prevalence of asthma amongst infants who were born with low birth weight and mothers didn’t smoke during pregnancy. To calculate the prevalence of asthma amongst normal birth weight and low birth weight infants whose mothers did smoke during pregnancy, we applied the odds ratio to the respective prevalence amongst infants whose mothers didn’t smoke using equation 4.

*Table C5.2: Odds ratios for increased asthma associated with maternal smoking within-pregnancy and exposure to passive smoking*

| **Age range (years)** | **Exposure: Maternal smoking during pregnancy** | | | **Exposure: Passive smoking from mother** | | |
| --- | --- | --- | --- | --- | --- | --- |
|  | **Mean** | **95% CI** | | **Mean** | **95% CI** | |
| **0-2** | 1.85 | 1.35 | 2.53 | 2.47 | 0.65 | 9.39 |
| **3-4** | 1.30 | 0.88 | 1.92 | 1.05 | 0.88 | 1.25 |
| **5-18** | 1.23 | 1.12 | 1.36 | 1.20 | 0.98 | 1.44 |
| **Source:** | Burke et al [30] | | | | | |

Once we had estimated the four prevalence’s:

*Prevalence (Asthma| Mother doesn’t smoke in pregnancy| Normal birth weight)*

*Prevalence (Asthma| Mother smokes in pregnancy| Normal birth weight)*

*Prevalence (Asthma| Mother doesn’t smoke in pregnancy| Low birth weight)*

*Prevalence (Asthma| Mother smokes in pregnancy| Low birth weight)*

We then calculated the prevalence controlling for whether the infant was exposed to passive smoking from the mother using the following equation:

$Prevalence \left( Asthma \right|Not passive smoking|Mother doesn^{'}t smoke in pregnancy |NBW)= \frac{Prevalence \left( Asthma \right|Mother doesn^{'}t smoke in pregnancy |NBW)}{(Prevalence(CS)\times{OR}_{PS})+Prevalence (NS)}$ (19)

Where $Prevalence \left( Asthma \right|Mother doesn^{'}t smoke in pregnancy |NBW)$ is the prevalence of asthma amongst those whose mothers didn’t smoke during pregnancy given being born normal bith weight irrespective on exposure to passive smoking, $Prevalence(CS)$ is the prevalence of smoking amongst mothers, $Prevalence (NS)$ is the prevalence of non-smoking amongst mothers (and hence is one minus $Prevalence\left( CS \right))$, and ${OR}_{PS}$ is the relative risk of increased asthma due to exposure to passive smoking. For the prevalence of smoking amongst mothers, we used 17.23 % (95% CI 15.97%-18.48%) from ONS data. [8] The odds ratios used can be found in Table C5.2. This process was repeated for the other three remaining prevalence’s until we had the four prevalence’s required:

*Prevalence (Asthma| Not passive smoking| Mother doesn’t smoke in pregnancy| Normal birth weight)*

*Prevalence (Asthma| Not passive smoking | Mother smokes in pregnancy| Normal birth weight)*

*Prevalence (Asthma| Not passive smoking | Mother doesn’t smoke in pregnancy| Low birth weight)*

*Prevalence (Asthma| Not passive smoking | Mother smokes in pregnancy| Low birth weight)*

To calculate the prevalence of asthma amongst those exposed to passive smoking, we then applied the odds ratios in Table C5.2 using equation 4. These values were bootstrapped 10,000 times to give us a mean and standard error for ESIP inputs, which can be found in Table 2 in Appendix D. For modelling simplicity, we used relative risks to capture the impact of passive smoking. These were estimated by dividing the Probability (Asthma| Exposed to passive smoking) by the Probability (Asthma | Not exposed to passive smoking), then weighting each relative risk by the prevalence of low/normal birth weight, and maternal within-pregnancy smoking behaviour, to give an average relative risk over all categories. The relative risks used as an input in ESIP can also be found in Table x in Appendix x.

*C6: Estimating ESIP inputs for maternal mortality during pregnancy*

To capture maternal morbidity associated with pregnancy, used ONS mortality statistics for England and Wales for 2006 to 2016 [31], which reported maternal deaths during pregnancy by morbidity. However, ONS estimates do not report the number of women giving birth by morbidity. Therefore, we used the HES data previously used to estimate the probability of morbidity as the number of women who had given birth in the year by morbidity. [13] We then divided the number of maternal deaths by the number of the number of women from the HES data which gave a per annum estimate of the probability of death for each morbidity / no morbidity. Because we assumed that smoking behaviour during pregnancy did not impact on mortality, we did not adjust these values to take into account within-pregnancy maternal smoking behaviour. Finally, we bootstrapped the per annum probabilities for each morbidity / no morbidity 10,000 times using the same process described above, estimating mean probabilities and their associated standard errors which were used in ESIP.

*C7: Incorporating the increased mortality associated with smoking into ESIP*

To capture the natural rate of death amongst the cohort of mothers and infants in the ‘lifetime’ components, data from ONS Cohort Life Tables was sought. [32] These give estimates of the probability of death by age from 1 years to 100 years, for cohorts of individuals born between 1981 and 2012, with projections up to cohorts born in 2062.^[[1]](#footnote-1)^ However, there is an increased risk of mortality associated with smoking behaviour (current and former smoker). To capture this, data was sought on the increased mortality associated with being a current and former smoker compared to a never smoker.

Doll et al reported mortality rates for a cohort of British male doctors. Mortality rates by smoking status can be found in Table C7.1.

*Table C7.1: Mortality rates amongst British male doctors per 1,000 people by age, contingent on smoking status*

| **Age range (years)** | **Current smokers** | **Former smokers** | **Never smokers** |
| --- | --- | --- | --- |
| **35-44** | 2.8 | 2 | 1.6 |
| **45-54** | 8.1 | 4.9 | 4 |
| **55-64** | 20.3 | 13.4 | 9.5 |
| **65-74** | 47 | 31.6 | 23.7 |
| **75-84** | 106 | 77.3 | 67.4 |
| **85+** | 218.7 | 179.7 | 168.6 |
| ***Source:*** | *Table 6, Doll et al [33, 34]* | | |

To apply this information to ESIP required the calculation of a relative risk for current smokers compared to never smokers and former smokers compared to never smokers. We estimated these relative risks by dividing the Current Smoker rate by the Never smoker rate. The estimated values used in ESIP can be found in Table 3 in Appendix D.

To apply these relative risks, ESIP performs the following calculation:

$$Probability\left( {Death| Never smoker}_{T} \right)$$

$= \frac{{Probability\left( Death \right|General population}_{T})}{\left( Prevalence\left( {Current smoker}_{T} \right)\times{RR}_{CS,T} \right)+\left( Prevalence\left( {Former Smoker}_{T} \right)\times{RR}_{FS,T} \right)+Prevalence ({Never Smokers}_{T})}$ (20)

Where ${Probability\left( Death \right|General population}_{T}$ is the probability of Death irrespective of smoking behaviour at age T, $Prevalence\left( {Current smoker}_{T} \right)$ is the prevalence of current smokers at age T, $Prevalence\left( {Former Smoker}_{T} \right)$ is the prevalence of former smokers at age T, $Prevalence ({Never Smokers}_{T})$ is the prevalence of never smokers at age T, ${RR}_{CS,T}$ is the relative risk for current smokers at age T, and ${RR}_{FS,T}$ is the relative risk for former smokers at age T. We used data from ONS estimates (see Table Adjfdf) for the prevalence of current, former, and never smokers by age. The ${Probability\left( Death \right|General population}_{T}$ came from the ONS Cohort Life Tables mentioned above, and are not reproduced here because they are based upon the birth year of the cohort.

*Appendix C8: Incorporating the increased mortality associated with low birth weight into the infant ‘childhood’ component*

LBW (infant being born weighing less than 2500 grams) is associated with an increased mortality amongst infants. Data was sought as to the impact of LBW has on mortality during childhood. The following data was identified:

*Table C8.1: Odds ratios for increased mortality during childhood associated with infants born with LBW compared to infants born with NBW*

| **Age range (years)** | **Odds ratio** | **95% CI** | |
| --- | --- | --- | --- |
| **1-4** | 2.2 | 1.9 | 2.5 |
| **5-9** | 1.7 | 1.3 | 2.1 |
| **10-14** | 1.5 | 1.1 | 1.9 |
| **15-19** | 0.9 | 0.7 | 1.1 |
| ***Source:*** | *Table 4, Li et al [35]* | | |

To be able to be applied to ESIP, these odds ratios needed recalculating into relative risks. We used the approach described by Grant [36], using the data reported in Table 4 of Li et al. [35] The estimated relative risks from this approach can be found in Table C3 in Appendix D. Relative risks where then applied to the ONS estimates of mortality (see section C7) using the following formula:

$Probability({Death| NBW}_{T})=\frac{{Probability (Death}_{T})}{\left( Prevalence\left( LBW \right)\times{RR}_{LBW. T} \right)+Prevalence(NBW)}$ (21)

Where ${Probability (Death}_{T})$ is the probability of death irrespective of birthweight, $Prevalence\left( LBW \right)$ is the prevalence of LBW infants at birth, $Prevalence(NBW)$ is the prevalence of NBW infants at birth (hence is one minus $Prevalence\left( LBW \right)$, and ${RR}_{LBW. T}$ is the relative risk of increased mortality associated with LBW at age T. We used the prevalence of LBW being 7.4337% (95% CI 7.3015%-7.5660%) from ONS data we had used in section C5 above. [29] The probability of death irrespective of birthweight came from the ONS Life Cohort Tables. [32] Thusly, the probability of death amongst LBW infants can be estimated by:

$Probability \left( {Death | LBW}_{T} \right)= Probability\left( {Death| NBW}_{T} \right)\times{RR}_{LBW. T}$ (22)

*C9: Fitting distributions to ESIP Inputs*

We used previously described methods for fitting distributions to ESIP inputs for the probabilistic sensitivity analyses. [21] Beta distributions were used for the probabilities of binary events (e.g. mother survives pregnancy or not) and for the utility decrements associated with foetal loss and ectopic pregnancy. Where events had more than two possible outcomes (e.g. a smoker stay smoking, try stopping, or die), the beta values were adjusted. This was done by summing all the sampled values for the respective node (decision tree) / state (Markov) and then dividing each value by the total, such that the values used in the iteration always summed to one. The normal distribution was used to sample utility tariffs, relative risks were sampled using the Log Normal distribution, and the Gamma distribution was used for sampling costs. Parameters for distributions were estimated from model inputs’ mean and standard errors where possible, however where there was no information regarding an inputs’ associated uncertainty (i.e. only the mean value was reported), we assumed the standard error to be that of 10% of the mean. [37]

*C10: Other limitations of ESIP*

Pre-term premature rupture of the membranes (PPROM) and congenital anomalies were identified by reviews as being smoking -related but were not incorporated into ESIP. We excluded PPROM because in most cases this results in normal births and so women experiencing PPROM would not be expected to incur substantial health care costs. [38] Congenital anomalies were not included because these have a low incidence [39] and are mostly minor; although some, relatively-infrequent major anomalies could be associated significant healthcare costs, the expected overall cost of these at a population level were anticipated to be very small. The omission of these conditions would suggest that ESIP underestimates the cost-savings associated with smoking cessation making estimates conservative.

It is possible that there are long-term morbidities caused by in-utero exposure to smoking or maternal smoking in the presence of children and these have not been included in ESIP due to a lack of evidence. Although we have identified and included all major morbidities currently associated with smoking in pregnancy from a review of the literature [40], others such as hypertension developing in the children of smokers when they reach adulthood [41]) may in future demonstrate a strong association with maternal smoking. However, as better evidence becomes available, ESIP could be modified to include such morbidities and so more accurately estimate gains in health and health care costs.

ESIP uses cross-sectional survey data to estimate smoking behaviour. These data are sampled for both genders across various age groups to provide probabilities for whether a never smoker will pick up smoking, for a current smoker to make a quit attempt, and whether a quitter will remain abstinent. Because the data are cross-sectional and not longitudinal, probabilities are calculated from respondents reporting smoking behaviour for different age groups in a particular year. Furthermore, the probabilities for successful quitting are calculated from data where respondents have had multiple quit attempts and hence are average probabilities of successfully stopping smoking. It is likely that these probabilities will need to be updated in the future, especially since current trends in smoking behaviour suggest that the proportion of “ever smokers” is in decline [8], and hence ESIP may be overestimating the smoking uptake amongst its infant cohort. Longitudinal cohort data would be preferable as this would demonstrate how individuals’ smoking behaviour changes over time; unfortunately no suitable longitudinal data were available. Therefore, the average probabilities are only an estimate of potential changes in smoking behaviour and an individual’s smoking behaviour changes may differ. Unfortunately, because of the absence of longitudinal data, we cannot determine the impact on using cross-sectional data for representing smoking behaviour, and therefore it is unclear what impact this may have on the model output. However, we deemed that since only cross-sectional data was available, it represented the best available data to allow us to estimate long term smoking behaviour.

ESIP uses data to estimate the increased mortality amongst current and former smokers (as compared to never smokers) based upon a study by Doll et al. [33, 34] This study is a longitudinal cohort study amongst British Male doctors, and as such may not represent the cohort of mothers and children represented in ESIP well. However, it has been used for similar recent decision analytic models [9, 42]. Therefore it was deemed as satisfactory for use in ESIP. AS more pertinent data becomes available, the authors will update ESIP accordingly so as to model the cohort of mothers and children as accurately as possible.

There is a significant literature on the levels of smoking and the increased risks of adverse events suggesting that there is a dose relationship between smoking and smoking associated morbidities. [43-46] ESIP does not take levels of smoking into account, focusing on the smoking behaviour of the cohort (current, former, or never smoker). This is partly due to model simplicity since the addition of levels of smoking would require additional states / pathways in the Markov models and Decision Trees. Furthermore, not all conditions have the relevant information regarding smoking dose and impact on morbidity, coupled with the extremely limited data on current smoker’s levels of smoking by age, which would have made including smoking levels impossible to include. While dose does seem an important consideration, ESIP still captures the majority of the impact that smoking has on the cohort, albeit it might be slightly over estimating the prevalence of morbidities due to assuming too many individuals have a higher level of smoking.

ESIP presents cost-effectiveness estimates for both deterministic and probabilistic analyses. While a deterministic analysis does present a single, easy to interpret figure, deterministic analyses are limited in that they do not take into account the impact of model input variability. [47] PSA is currently the standard approach for demonstrating the impact of uncertainty on model outputs and this is why it was incorporated into ESIP. [21, 37, 48] With deterministic analyses, decisions made using model outputs upon what could be considered based on ‘one-off’ finding due to the selection of model inputs on that particular day. Although one-way sensitivity analyses have their uses for demonstrating the impact of varying particular inputs on the result of the evaluation [21], these also give an incomplete picture of how uncertainty might impact on model outputs. [47] Therefore, the currently accepted approach is to allow all model inputs to vary simultaneously. [21, 37, 48] This requires that all model inputs have a suitable distribution fitted so that ESIP can sample relevant values based on the uncertainty (usually informed by the standard error of the mean input value or the 95% confidence interval) associated with each model input. Because of the large number of model inputs in ESIP, we felt it was necessary to do 10,000 simulations to get an accurate picture of the impact associated with input uncertainty. However, to enable a PSA to be conducted, we require further assumptions about the model inputs, such as the uncertainty associated with input, the type of distribution fitted, and that we assume that all model inputs are independent (i.e. a change in value for one input will not impact on the sampled value for another input). [49] Therefore, although the PSA might not be estimating the true impact of model input uncertainty on outputs, it does this more comprehensively than one-way sensitivity analyses do.

**Appendix D: Prevalence of Morbidities and Mortality**

*Table D1: ESIP inputs for prevalence of morbidities and associated RR for former and current smokers for maternal ‘lifetime’ and infant ‘adulthood’ components, as estimated by the process described in Section C4 in Appendix C.*

| **Condition & age range (years)** | **Females** | | | | | | | | **Males** | | | | | | | |
| --- | --- | --- | --- | --- | --- | --- | --- | --- | --- | --- | --- | --- | --- | --- | --- | --- |
|  | **Prevalence amongst never smokers** | | **RR for former smokers*** | | | **RR for current smokers*** | | | **Prevalence amongst never smokers** | | **RR for former smokers*** | | | **RR for current smokers*** | | |
|  | **Mean** | **SE** | **Mean** | **95% CI** | | **Mean** | **95% CI** | | **Mean** | **SE** | **Mean** | **95% CI** | | **Mean** | **95% CI** | |
| **CHD** | | | | | | | | | | | | | | | | |
| 0-15 | 0 | 0 | 1 | | | 1 | | | 0 | 0 | 1 | | | 1 | | |
| 16-24 | .0010 | .0001 |  |  |  |  |  |  | .0010 | .0001 |  |  |  |  |  |  |
| 25-34 | .0010 | .0001 |  |  |  |  |  |  | .0020 | .0002 |  |  |  |  |  |  |
| 35-44 | .0015 | .0002 | 2.23 | 1.79 | 2.67 | 4.98 | 4.00 | 5.96 | .0033 | .0004 | 1.83 | 1.47 | 2.19 | 3.88 | 3.12 | 4.64 |
| 45-49 | .0064 | .0007 |  |  |  |  |  |  | .0195 | .0022 |  |  |  |  |  |  |
| 50-54 | .0066 | .0008 |  |  |  |  |  |  | .0203 | .0023 |  |  |  |  |  |  |
| 55-59 | .0241 | .0027 | 1.21 | 0.97 | 1.45 | 3.25 | 2.61 | 3.89 | .0696 | .0078 | 1.52 | 1.22 | 1.82 | 2.99 | 2.40 | 3.58 |
| 60-64 | .0301 | .0050 |  |  |  |  |  |  | .0722 | .0083 |  |  |  |  |  |  |
| 65-69 | .0778 | .0114 | 1.56 | 1.25 | 1.87 | 3.29 | 2.65 | 3.93 | .1419 | .0160 | 1.58 | 1.27 | 1.89 | 2.76 | 2.22 | 3.30 |
| 70-74 | .0776 | .0118 |  |  |  |  |  |  | .1419 | .0159 |  |  |  |  |  |  |
| 75-79 | .1613 | .0196 | 1.27 | 1.02 | 1.52 | 2.25 | 1.81 | 2.69 | .2271 | .0255 | 1.32 | 1.06 | 1.58 | 1.98 | 1.59 | 2.37 |
| 80-84 | .1610 | .0197 |  |  |  |  |  |  | .2270 | .0253 |  |  |  |  |  |  |
| 85+ | .1611 | .0199 |  |  |  |  |  |  | .2269 | .0256 |  |  |  |  |  |  |
| **COPD** | | | | | | | | | | | | | | | | |
| 0-4 | .0001 | .0000 | 1 | | | 1 | | | 0 | 0 | 1 | | | 1 | | |
| 5-9 | .0001 | .0000 |  |  |  |  |  |  | .0001 | .0000 |  |  |  |  |  |  |
| 10-14 | .0002 | .0000 |  |  |  |  |  |  | .0002 | .0000 |  |  |  |  |  |  |
| 15-19 | .0003 | .0000 |  |  |  |  |  |  | .0003 | .0000 |  |  |  |  |  |  |
| 20-24 | .0001 | .0000 |  |  |  |  |  |  | .0003 | .0000 |  |  |  |  |  |  |
| 25-29 | .0002 | .0000 |  |  |  |  |  |  | .0004 | .0000 |  |  |  |  |  |  |
| 30-34 | .0004 | .0000 |  |  |  |  |  |  | .0005 | .0000 |  |  |  |  |  |  |
| 35-39 | .0004 | .0000 | 1.85 | 1.49 | 2.21 | 6.43 | 5.17 | 7.69 | .0004 | .0001 | 2.22 | 1.78 | 2.66 | 4.47 | 3.59 | 5.35 |
| 40-44 | .0010 | .0001 |  |  |  |  |  |  | .0011 | .0001 |  |  |  |  |  |  |
| 45-49 | .0024 | .0003 |  |  |  |  |  |  | .0023 | .0003 |  |  |  |  |  |  |
| 50-54 | .0052 | .0006 |  |  |  |  |  |  | .0050 | .0006 |  |  |  |  |  |  |
| 55-59 | .0061 | .0007 | 4.84 | 3.89 | 5.79 | 9.00 | 7.24 | 10.76 | .0045 | .0006 | 3.98 | 3.20 | 4.76 | 15.17 | 12.20 | 18.14 |
| 60-64 | .0118 | .0020 |  |  |  |  |  |  | .0086 | .0010 |  |  |  |  |  |  |
| 65-69 | .0063 | .0014 | 15.72 | 12.64 | 18.80 | 38.89 | 31.27 | 46.51 | .0077 | .0010 | 8.13 | 6.54 | 9.72 | 29.69 | 23.87 | 35.51 |
| 70-74 | .0077 | .0018 |  |  |  |  |  |  | .0104 | .0013 |  |  |  |  |  |  |
| 75-79 | .0180 | .0042 | 7.06 | 5.68 | 8.44 | 20.96 | 16.85 | 25.07 | .0154 | .0019 | 6.55 | 5.27 | 7.836 | 23.01 | 18.50 | 27.52 |
| 80-84 | .0179 | .0043 |  |  |  |  |  |  | .0173 | .0021 |  |  |  |  |  |  |
| 85+ | .0140 | .0034 |  |  |  |  |  |  | .0162 | .0020 |  |  |  |  |  |  |
| **Lung Cancer** | | | | | | | | | | | | | | | | |
| 0-34 | .0000 | .0000 | 1 | | | 1 | | | .0000 | .0000 | 1 | | | 1 | | |
| 35-44 | .0000 | .0000 | 2.64 | 2.12 | 3.16 | 13.30 | 10.69 | 15.91 | .0000 | .0000 | 4.40 | 3.54 | 5.26 | 14.33 | 11.52 | 17.14 |
| 45-54 | .0002 | .0000 |  |  |  |  |  |  | .0003 | .0000 |  |  |  |  |  |  |
| 55-59 | .0001 | .0000 | 5.00 | 4.02 | 5.98 | 18.95 | 15.24 | 22.66 | .0003 | .0000 | 4.57 | 3.67 | 5.47 | 19.03 | 15.30 | 22.76 |
| 60-64 | .0002 | .0001 |  |  |  |  |  |  | .0003 | .0000 |  |  |  |  |  |  |
| 65-74 | .0006 | .0002 | 6.80 | 5.47 | 8.13 | 23.65 | 19.01 | 28.29 | .0011 | .0001 | 7.79 | 6.26 | 9.32 | 28.29 | 22.75 | 33.83 |
| 75+ | .0006 | .0002 | 6.38 | 5.13 | 7.63 | 23.08 | 18.56 | 27.60 | .0013 | .0002 | 6.46 | 5.19 | 7.73 | 22.51 | 18.10 | 26.92 |
| **Stoke** | | | | | | | | | | | | | | | | |
| 0-15 | 0 | 0 | 1 | | | 1 | | | 0 | 0 | 1 | | | 1 | | |
| 16-24 | .0020 | .0002 |  |  |  |  |  |  | 0 | 0 |  |  |  |  |  |  |
| 25-34 | .0010 | .0001 |  |  |  |  |  |  | 0 | 0 |  |  |  |  |  |  |
| 35-44 | .0031 | .0003 | 1.00 | 0.80 | 1.20 | 2.44 | 1.96 | 2.92 | .0038 | .0004 | 1.07 | 0.86 | 1.28 | 2.40 | 1.93 | 2.87 |
| 45-49 | .0070 | .0008 |  |  |  |  |  |  | .0090 | .0010 |  |  |  |  |  |  |
| 50-54 | .0071 | .0008 |  |  |  |  |  |  | .0093 | .0010 |  |  |  |  |  |  |
| 55-59 | .0192 | .0020 | 1.10 | 0.88 | 1.32 | 1.98 | 1.59 | 2.37 | .0210 | .0023 | 1.51 | 1.21 | 1.81 | 2.51 | 2.02 | 3.00 |
| 60-64 | .0214 | .0027 |  |  |  |  |  |  | .0214 | .0025 |  |  |  |  |  |  |
| 65-69 | .0369 | .0048 | 1.24 | 1.00 | 1.48 | 2.27 | 1.83 | 2.71 | .0570 | .0064 | 1.23 | 0.99 | 1.47 | 2.17 | 1.74 | 2.60 |
| 70-74 | .0369 | .0048 |  |  |  |  |  |  | .0571 | .0062 |  |  |  |  |  |  |
| 75-79 | .1002 | .0117 | 1.10 | 0.88 | 1.32 | 1.70 | 1.37 | 2.03 | .1178 | .0130 | 1.12 | 0.90 | 1.34 | 1.48 | 1.19 | 1.77 |
| 80-84 | .1005 | .0120 |  |  |  |  |  |  | .1177 | .0129 |  |  |  |  |  |  |
| 85+ | .1004 | .0119 |  |  |  |  |  |  | .1180 | .0131 |  |  |  |  |  |  |

*Table D2: ESIP inputs for the prevalence of asthma amongst infants with non-smoking mothers and associated RR for exposure to passive smoking as used in the infant ‘childhood’ component, as estimated in the process described in section C5 in Appendix C*

| **Birthweight, within-pregnancy smoking behaviour, & age range (years)** | **Females** | | | | | **Males** | | | | |
| --- | --- | --- | --- | --- | --- | --- | --- | --- | --- | --- |
|  | **Prevalence amongst infants with abstinent mothers** | | **RR for exposure to passive smoking** | | | **Prevalence amongst infants with abstinent mothers** | | **RR for exposure to passive smoking** | | |
|  | **Mean** | **SE** | **Mean** | **95% CI** | | **Mean** | **SE** | **Mean** | **95% CI** | |
| **LBW & Mother abstinent at delivery** | | | | | | | | | | |
| 1-2 | .0122 | .0031 | 3.02 | .65 | 8.63 | .0205 | .0052 | 2.97 | .66 | 8.34 |
| 3-4 | .0163 | .0021 | 1.05 | .88 | 1.25 | .0375 | .0103 | 1.20 | .94 | 1.54 |
| 5-9 | .0830 | .0102 | 1.19 | .99 | 1.42 | .1257 | .0152 | 1.18 | .99 | 1.39 |
| 10-14 | .1909 | .0215 | 1.17 | .99 | 1.38 | .2524 | .0272 | 1.17 | .99 | 1.36 |
| 15-19 | .2433 | .0260 | 1.15 | .99 | 1.33 | .2907 | .0306 | 1.14 | .99 | 1.31 |
| **LBW & Mother smoking at delivery** | | | | | | | | | | |
| 1-2 | .0224 | .0063 | As above | | | .0375 | .0103 | As above | | |
| 3-4 | .0214 | .0045 |  |  |  | .0358 | .0074 |  |  |  |
| 5-9 | .0933 | .0118 |  |  |  | .1733 | .0202 |  |  |  |
| 10-14 | .2224 | .0251 |  |  |  | .2895 | .0309 |  |  |  |
| 15-19 | .2807 | .0296 |  |  |  | .3313 | .0341 |  |  |  |
| **NBW & Mother abstinent at delivery** | | | | | | | | | | |
| 1-2 | .0095 | .0023 | As above | | | .0160 | .0039 | As above | | |
| 3-4 | .0128 | .0014 |  |  |  | .0215 | .0023 |  |  |  |
| 5-9 | .0556 | .0061 |  |  |  | .1063 | .0110 |  |  |  |
| 10-14 | .0956 | .0107 |  |  |  | .0944 | .0118 |  |  |  |
| 15-19 | .2013 | .0197 |  |  |  | .2434 | .0238 |  |  |  |
| **NBW & Mother smoking at delivery** | | | | | | | | | | |
| 1-2 | .0176 | .0047 | As above | | | .0295 | .0079 | As above | | |
| 3-4 | .0168 | .0033 |  |  |  | .0282 | .0055 |  |  |  |
| 5-9 | .0794 | .0088 |  |  |  | .1349 | .0143 |  |  |  |
| 10-14 | .1855 | .0185 |  |  |  | .2462 | .0241 |  |  |  |
| 15-19 | .2369 | .0233 |  |  |  | .2839 | .0274 |  |  |  |

*Table D3: ESIP inputs for estimates of relative risks for increased mortality associated with smoking behaviour (compared to never smokers) and LBW (compared to NBW) as used in the maternal ‘lifetime’ and infant ‘childhood’ and ‘adulthood’ components, as described in section C7 and C8 in Appendix C*

| **Smoking behaviour/birth weight & age range (years)** | **RR for increased mortality** | | |
| --- | --- | --- | --- |
|  | **Mean** | **95% CI** | |
| **Former smokers** | | | |
| 0-35 | 1 | | |
| 35-44 | 1.25 | 1.01 | 1.50 |
| 45-54 | 1.23 | .98 | 1.47 |
| 55-64 | 1.41 | 1.13 | 1.69 |
| 65-74 | 1.33 | 1.07 | 1.59 |
| 75-84 | 1.15 | .92 | 1.37 |
| 85+ | 1.07 | .86 | 1.27 |
| **Current smokers** | | | |
| 0-35 | 1 | | |
| 35-44 | 1.75 | 1.41 | 2.09 |
| 45-54 | 2.03 | 1.63 | 2.42 |
| 55-64 | 2.14 | 1.72 | 2.56 |
| 65-74 | 1.98 | 1.59 | 2.37 |
| 75-84 | 1.57 | 1.26 | 1.88 |
| 85+ | 1.30 | 1.04 | 1.55 |
| **LBW (compared to NBW)** | | | |
| 1-4 | 2.01 | 1.77 | 2.24 |
| 5-9 | 1.66 | 1.29 | 2.02 |
| 10-14 | 1.48 | 1.10 | 1.85 |
| 15-19 | .91 | .72 | 1.09 |

***References***

1. Jones, M., et al., *Re-starting smoking in the postpartum period after receiving a smoking cessation intervention: a systematic review.* Addiction, 2016: p. n/a-n/a.

2. McAndrew, F., et al., *Infant Feeding Survey 2010*, The NHS Information Centre for health and social care, Editor. 2012, Health and Social Care Information Centre: London.

3. Lifestyle Statistics and Health and Social Care Information Centre, *Statistics of Smoking: England 2013*. 2013: London.

4. Yudkin, P., et al., *Abstinence from smoking eight years after participation in randomised controlled trial of nicotine patch.* BMJ, 2003. **327**(7405): p. 28-9.

5. Information Centre for Health and Social Care and Department of Health, *Health Survey for England*. 2017, UK Data Service: London.

6. Fleurence, R.L. and C.S. Hollenbeak, *Rates and probabilities in economic modelling: transformation, translation and appropriate application.* Pharmacoeconomics, 2007. **25**(1): p. 3-6.

7. DiFranza, J.R. and R.A. Lew, *Effect of maternal cigarette smoking on pregnancy complications and sudden infant death syndrome.* J Fam Pract, 1995. **40**(4): p. 385-94.

8. Office for National Statistics, *Adult Smoking Habits in Great Britian*. 2017, Office for National Statistics: London.

9. Flack, S., M. Taylor, and P. Trueman, *Cost-Effectivness of Interventions for Smoking Cessation*, in *Final Report*, National Institute for Health and Care Excellence, Editor. 2007, York Health Economics Consortium: York.

10. Leonardi-Bee, J., M.L. Jere, and J. Britton, *Exposure to parental and sibling smoking and the risk of smoking uptake in childhood and adolescence: a systematic review and meta-analysis.* Thorax, 2011. **66**(10): p. 847-55.

11. Mennemeyer, S.T. and L.P. Cyr, *A bootstrap approach to medical decision analysis.* J Health Econ, 1997. **16**(6): p. 741-7.

12. Glick, H.A., et al., *Economic evaluation in clinical trials*. 2014: OUP Oxford.

13. Health and Social Care Information Centre, *Hospital Episode Statistics: NHS Maternity Statistics - England*. 2015, Health and Social Care Information Centre,.

14. Office for National Statistics. *Gestation-specific infant mortality in England and Wales*. 2015 15th October 2014 [cited 2015 15th September]; Available from: <http://www.ons.gov.uk/ons/rel/child-health/gestation-specific-infant-mortality-in-england-and-wales/index.html>.

15. Office for National Statistics. *Child mortality statistics: Childhood, infant and perinatal*. 2014 30th January 2014 [cited 2014 10th June]; Available from: <http://www.ons.gov.uk/ons/rel/vsob1/child-mortality-statistics--childhood--infant-and-perinatal/index.html>.

16. Office for National Statistics, *Mortality Statistics: Childhood, infant and perinatal, England and Wales (Series DH3) - No. 39, 2006*. 2008, Office for National Statistics,.

17. Office for National Statistics, *Mortality Statistics: Childhood, infant and perinatal, England and Wales (Series DH3) - No. 40, 2007*. 2009, Office for National Statistics,.

18. StataCorp, *Stata /SE 15.1*. 2017, StataCorp LP,: College Station, TX.

19. Castles, A., et al., *Effects of smoking during pregnancy. Five meta-analyses.* American journal of preventive medicine, 1999. **16**(3): p. 208-15.

20. Shah, N.R. and M.B. Bracken, *A systematic review and meta-analysis of prospective studies on the association between maternal cigarette smoking and preterm delivery.* Am J Obstet Gynecol, 2000. **182**(2): p. 465-72.

21. Briggs, A., K. Claxton, and M. Sculpher, *Decision Modelling for Health Economic Evaluation*. Handbooks in Health Economic Evaluation. 2006, Oxford: Oxford University Press.

22. Health and Social Care Information Centre, *Statistics on Women's Smoking Status at Time of Delivery: England*, L. Statistics, Editor. 2016, Office for National Statistics: London.

23. Townsend, N., et al., *Coronary heart disease statistics: A compendium of health statistics: 2012 edition*. 2012, Department of Public Health, University of Oxford,: London.

24. Flowers, J., et al., *Chronic disease prevalence by age, sex and region in 2008*, Eastern Region Public Health Observatory (ERPHO), Editor. 2010, Public Health England,: London.

25. Forman, D., et al., *Cancer prevalence in the UK: results from the EUROPREVAL study.* Ann Oncol, 2003. **14**(4): p. 648-54.

26. U.S. Department of Health and Human Services, *The Health Consequences of Smoking—50 Years of Progress. A Report of the Surgeon General*, Department of Health and Human Services, Editor. 2014, Centers for Disease Control and Prevention, National Center for Chronic Disease Prevention and Health Promotion, Office on Smoking and Health,: Atlanta, GA: US.

27. Mu, M., et al., *Birth Weight and Subsequent Risk of Asthma: A Systematic Review and Meta-Analysis.* Heart, Lung and Circulation, 2014(0).

28. Royal College of Physicians, *Passive smoking and children. A report by the Tobacco Advisory Group.* 2010, RCP: London.

29. Office for National Statistics. *Child mortality statistics: Childhood, infant and perinatal*. 2015 10th March 2015 [cited 2015 17th September]; Available from: <http://www.ons.gov.uk/ons/rel/vsob1/child-mortality-statistics--childhood--infant-and-perinatal/index.html>.

30. Burke, H., et al., *Prenatal and Passive Smoke Exposure and Incidence of Asthma and Wheeze: Systematic Review and Meta-analysis.* Pediatrics, 2012. **129**(4): p. 735-744.

31. Office for National Statistics, *Mortality Statistics: Death Registered in England and Wales (Series DR)*, Office for National Statistics, Editor. 2008-2016: London.

32. Office for National Statistics, *Historic and Projected Mortality Data from the Period and Cohort Life Tables, 2012-based, UK, 1981-2062*. 2013, Office for National Statistics,: London.

33. Doll, R., et al., *Mortality in relation to smoking: 40 years' observations on male British doctors.* BMJ, 1994. **309**(6959): p. 901-911.

34. Doll, R., et al., *Mortality in relation to smoking: 50 years' observations on male British doctors.* BMJ, 2004. **328**(7455): p. 1519.

35. Li, C.I., J.R. Daling, and I. Emanuel, *Birthweight and risk of overall and cause-specific childhood mortality.* Paediatr Perinat Epidemiol, 2003. **17**(2): p. 164-70.

36. Grant, R.L., *Converting an odds ratio to a range of plausible relative risks for better communication of research findings.* BMJ : British Medical Journal, 2014. **348**.

37. Husereau, D., et al., *Consolidated Health Economic Evaluation Reporting Standards (CHEERS)--explanation and elaboration: a report of the ISPOR Health Economic Evaluation Publication Guidelines Good Reporting Practices Task Force.* Value Health, 2013. **16**(2): p. 231-50.

38. National Institute for Health and Care Excellence. *NICE Pathways: Suspected or certain prelabour rupture of the membranes at term*. 2014 2007 [cited 2014 6th August]; Available from: <http://pathways.nice.org.uk/pathways/intrapartum-care#path=view%3A/pathways/intrapartum-care/prelabour-rupture-of-the-membranes-at-term.xml&content=view-node%3Anodes-suspected-or-certain-prelabour-rupture-of-the-membranes-at-term>.

39. European Surveillance of Congenital Anomalies. *EUROCAT Prevalence Tables*. 2013 [cited 2013 31/12/2013]; Available from: <http://www.eurocat-network.eu/accessprevalencedata/prevalencetables>.

40. Jones, M.J., *The development of the Economic impacts of Smoking In Pregnancy (ESIP) model for measuring the impacts of smoking and smoking cessation during pregnancy*, in *School of Medicine*. 2015, University of Nottingham: Nottingham. p. 496.

41. Gao, Y.J., et al., *Effects of fetal and neonatal exposure to nicotine on blood pressure and perivascular adipose tissue function in adult life.* Eur J Pharmacol, 2008. **590**(1-3): p. 264-8.

42. Taylor, M., *Economic Analysis of Interventions for Smoking Cessation Aimed at Pregnant Women*, in *NICE Guidance PH26, Supplementary Report*, National Institute for Health and Care Excellence, Editor. 2009, York Health Economics Consortium.

43. Law, M.R., et al., *The dose-response relationship between cigarette consumption, biochemical markers and risk of lung cancer.* British Journal of Cancer, 1997. **75**(11): p. 1690-1693.

44. Ellard, G.A., et al., *Smoking during pregnancy: the dose dependence of birthweight deficits.* Br J Obstet Gynaecol, 1996. **103**(8): p. 806-13.

45. Centers for Disease Control and Prevention (US), National Center for Chronic Disease Prevention and Health Promotion (US), and Office on Smoking and Health (US), *How Tobacco Smoke Causes Disease: The Biology and Behavioral Basis for Smoking-Attributable Disease: A Report of the Surgeon General.* Vol. 6. 2010, Atlanta (GA): Centers for Disease Control and Prevention (US).

46. Marufu, T.C., et al., *Maternal smoking and the risk of still birth: systematic review and meta-analysis.* BMC Public Health, 2015. **15**(1): p. 239.

47. Claxton, K., et al., *Probabilistic sensitivity analysis for NICE technology assessment: not an optional extra.* Health Econ, 2005. **14**(4): p. 339-47.

48. National Institute for Health and Care Excellence. *Guide to the methods of technology appraisal 2013*. 2013 04/05/2013 [cited 2014 15th May]; Available from: <http://publications.nice.org.uk/guide-to-the-methods-of-technology-appraisal-2013-pmg9/the-reference-case>.

49. O'Brien, B.J., et al., *In search of power and significance: issues in the design and analysis of stochastic cost-effectiveness studies in health care.* Med Care, 1994. **32**(2): p. 150-63.

1. Note: due to space constraints, these values are not reproduced in this Appendix. Please go directly to the ONS life tables which can be found at: http://www.ons.gov.uk/ons/rel/lifetables/historic-and-projected-data-from-the-period-and-cohort-life-tables/2012-based/stb-2012-based.html [↑](#footnote-ref-1)
